# Supplementary material for: Identification and characterization of Varicella Zoster Virus circular RNA in lytic infection
Source: Nat Commun. 2024 Jun 10;15:4932. doi: 10.1038/s41467-024-49112-4 (PMC11164961; doi:10.1038/s41467-024-49112-4)
Supplement: Supplementary file 1 — Supplementary Information [file 41467_2024_49112_MOESM1_ESM.pdf]

Supplementary Information

Title: Identification and Characterization of Varicella Zoster Virus Circular RNA in Lytic Infection

Authors: Shaomin Yang[1,2†], Di Cao[1†], Dabbu Kumar Jaijyan[3 †], Mei Wang [4], Jian Liu[5], Ruth Cruz-cosme[6], Songbin Wu[1], Jiabin Huang[1], Mulan Zeng[3], Xiaolian Liu [4], Wuping Sun[1], Donglin Xiong[1], Qiyi Tang[6*], Lizu Xiao[1*], Hua Zhu[3*]

Affiliations:

[1] Department of Pain Medicine and Shenzhen Municipal Key Laboratory for Pain Medicine, Huazhong University of Science and Technology Union Shenzhen Hospital, Shenzhen, China.

[2] Guangdong Key Laboratory for Biomedical Measurements and Ultrasound Imaging, National-Regional Key Technology Engineering Laboratory for Medical Ultrasound, School of Biomedical Engineering, Shenzhen University Medical School, Shenzhen 518060, China.

[3] Department of Microbiology and Molecular Genetics, New Jersey Medical School, Rutgers University, 225 Warren Street, Newark, NJ 070101, USA.

[4] Institute of Medical Microbiology, Jinan University, Guangzhou, Guangdong, 510632, China.

[5] School of Biological Sciences and Biotechnology, Minnan Normal University, Zhangzhou 363000, China.

[6] Department of Microbiology, Howard University College of Medicine, 520 W Street NW Washington, DC 20059, USA.

† These authors contributed equally.

[*] These authors jointly supervised this work: qiyi.tang@howard.edu, nsyyjoe@live.cn and hua.zhu@rutgers.edu

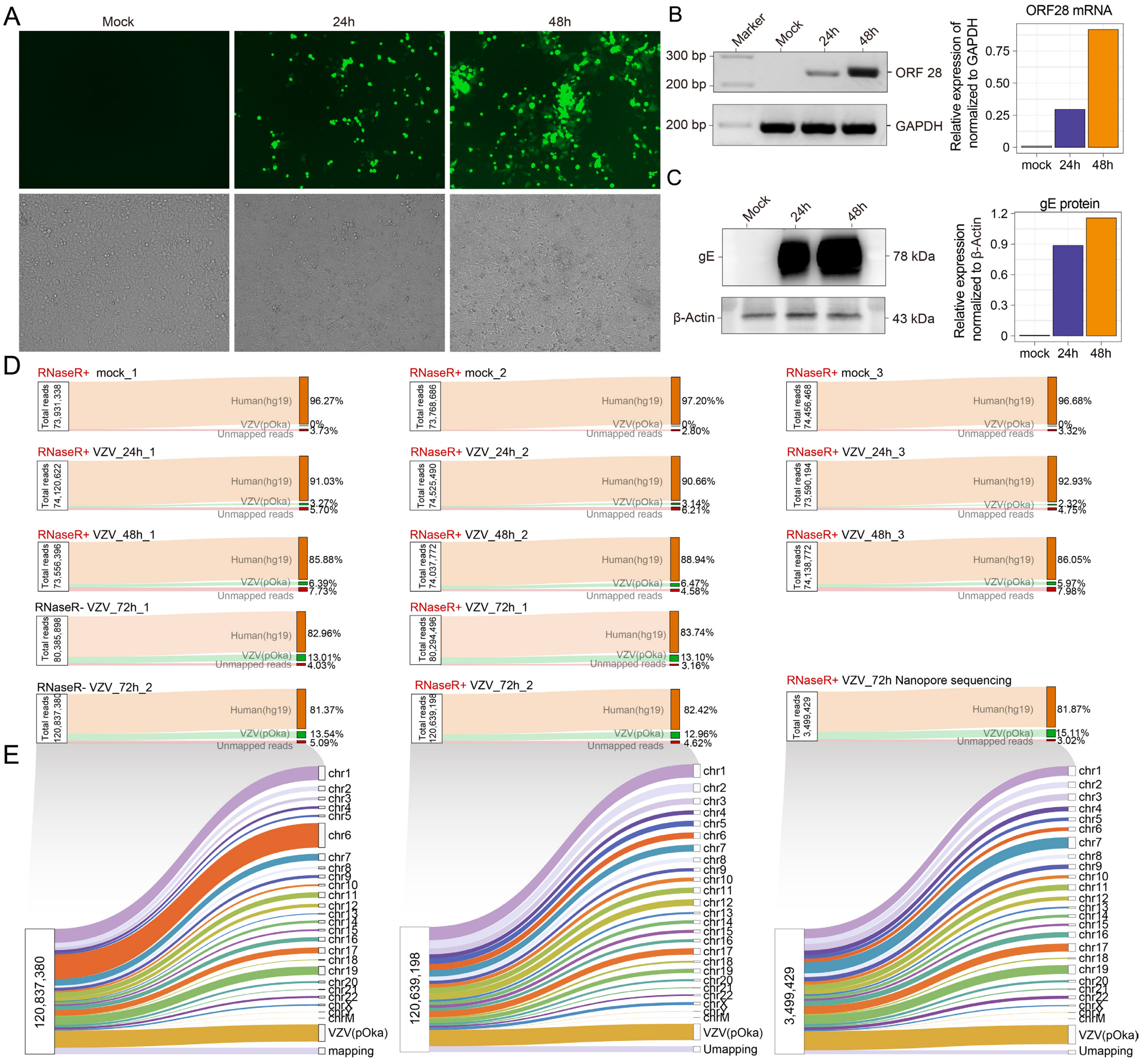

Supplementary Figure 1. SH-SY5Y cells infected with VZV obvious cytopathic changes and RNA-seq mapping statistics.

(A) SH-SY5Y cells infected with VZV pOka for 24h, 48 h. Cells harboring VZV are GFP-positive. **(B)** RT-PCR was used to measure the expression of ORF28. The right panel shows the relative expression of bands. **(C)** Western blot was used to evaluate the expression of VZV gE glycoprotein (Abcam, Cat. no. ab272686, which was used at a concentration of 1 μg/mL). **(D)** Mapping statistics of total read mapping to human or VZV genome and (E) each chromosome. Source data are provided as a Source Data file.

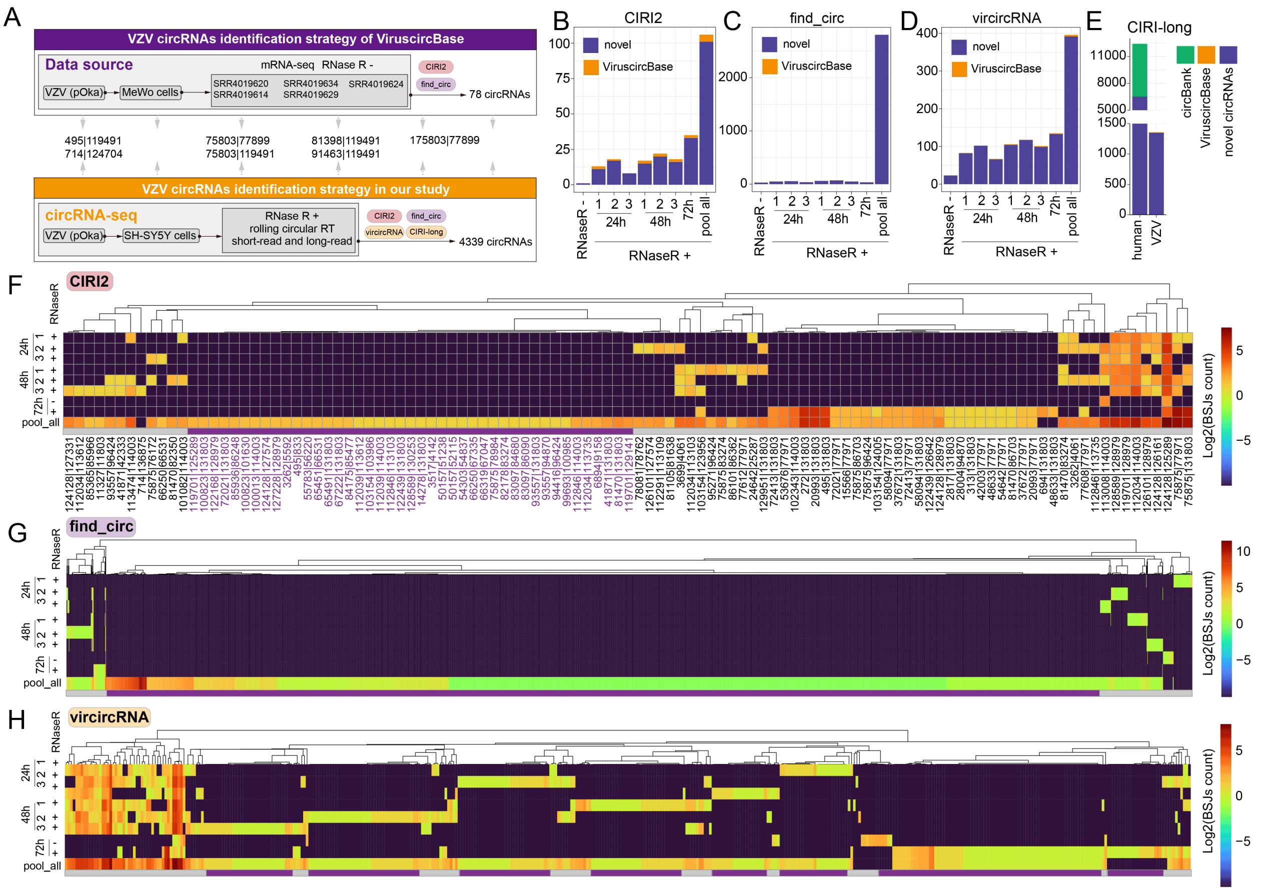

Supplementary Figure 2. Statistics of VZV circRNAs. (A) Schematic diagram of VZV circRNA identification strategy of ViruscircBase and this study. **(B-E)** Statistics of CIRI2 (B), find_circ (C), vircircRNA (D) and CIRI-long (E) identified VZV circRNAs in each group. **(F-H)** Heatmap of CIRI2 (F), find_circ (G) and vircircRNA(H) identified VZV circRNAs from each sample or pooled biological replicates as one sample. Source data are provided as Supplementary data 1 and Supplementary data 2.

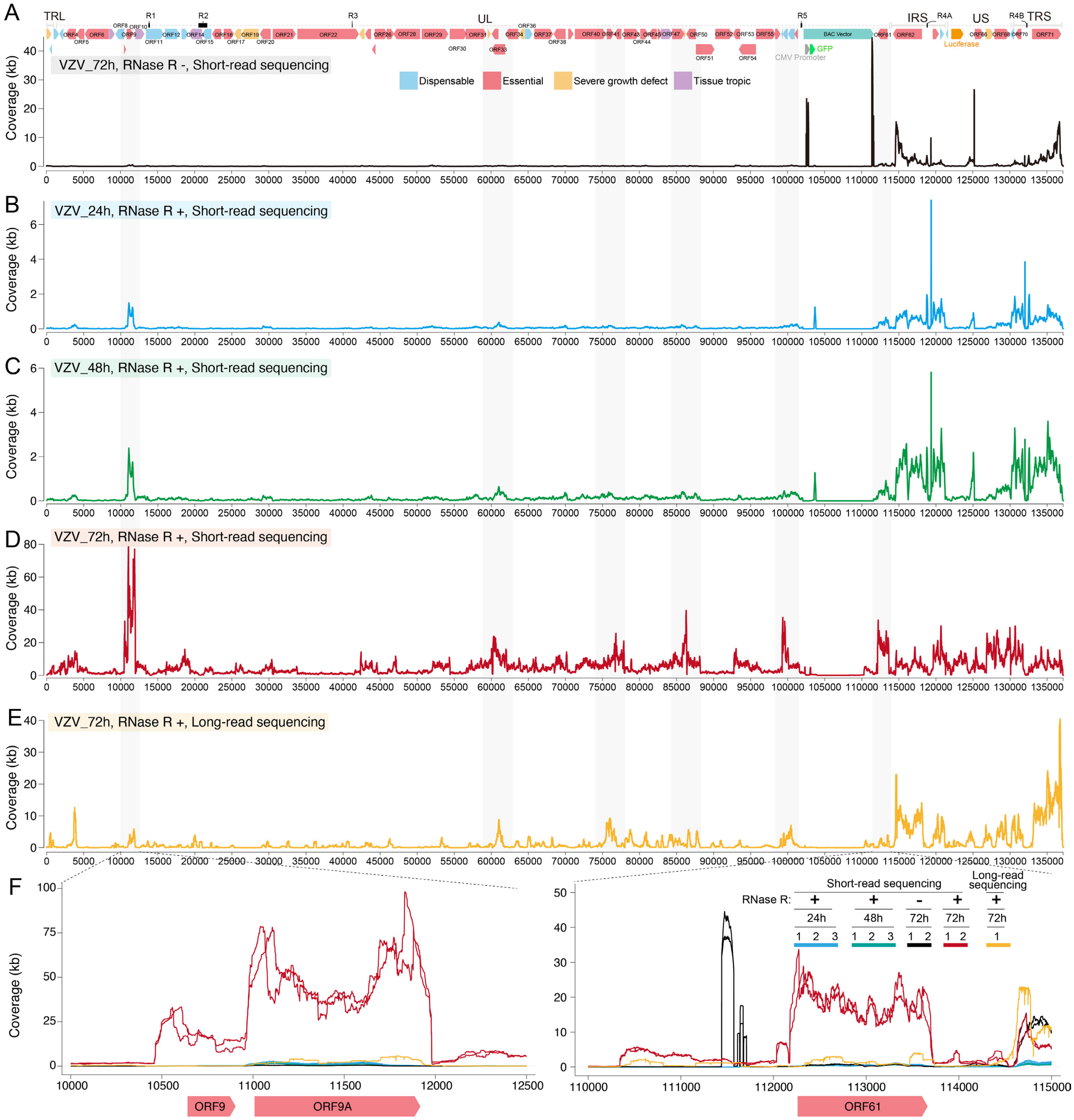

Supplementary Figure 3. The comparison of VZV RNA genome coverage level.

(A-F) Genome coverage of VZV RNA mapping to pOka strain with GFP and luciferase gene (GenBank accession number, PP054841, https://www.ncbi.nlm.nih.gov/nuccore/PP054841). VZV infected 24h (A), 48h (B) with RNase R treatment; VZV infected 72h with (C) or without (D) RNase R treatment, using BGISEQ short-read sequencing. (E) VZV infected 72h using ONT long-read sequencing with RNase R treatment. (F) The coverage in specific location of ORF9,

ORF9A and ORF61. The ORFs, unique and the repeat regions (TRL, IRL, UL, IRS, US, TRS) and multiple short reiterations regions (R1, R2, R3, R4, R5) in loci on the VZV genome map were included[1]. The ORFs are were color-coded according to the growth properties of their corresponding virus gene-deletion mutants[2]. Source data are provided as a Source Data file.

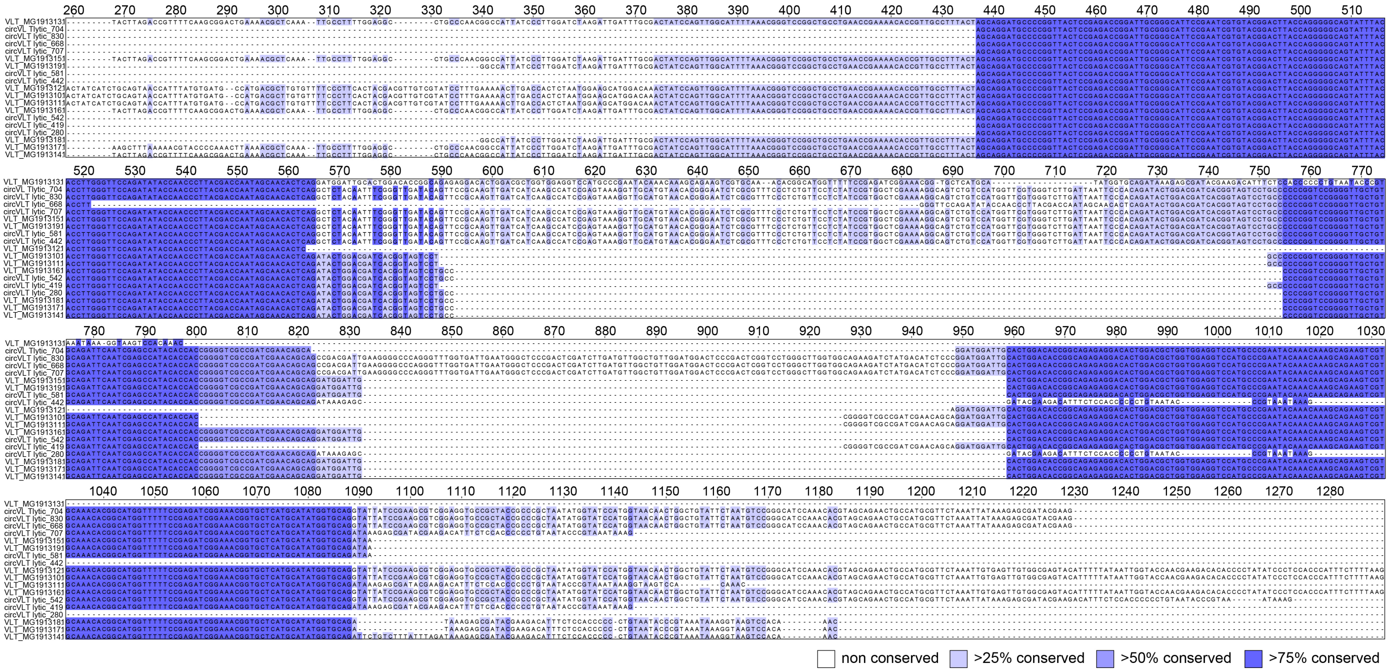

Supplementary Figure 4. Sequence and structural alignment of VLTs and VZV circVLTs$_{lytic}$. VLTs sequences (MG191310.1, MG191311.1, MG191312.1, MG191313.1, MG191314.1, MG191315.1, MG191316.1, MG191317.1, MG191318.1, MG191319.1) were obtained from NCBI[3] and aligned to VZV circVLTs isoforms. Source data are provided as a Source Data file.

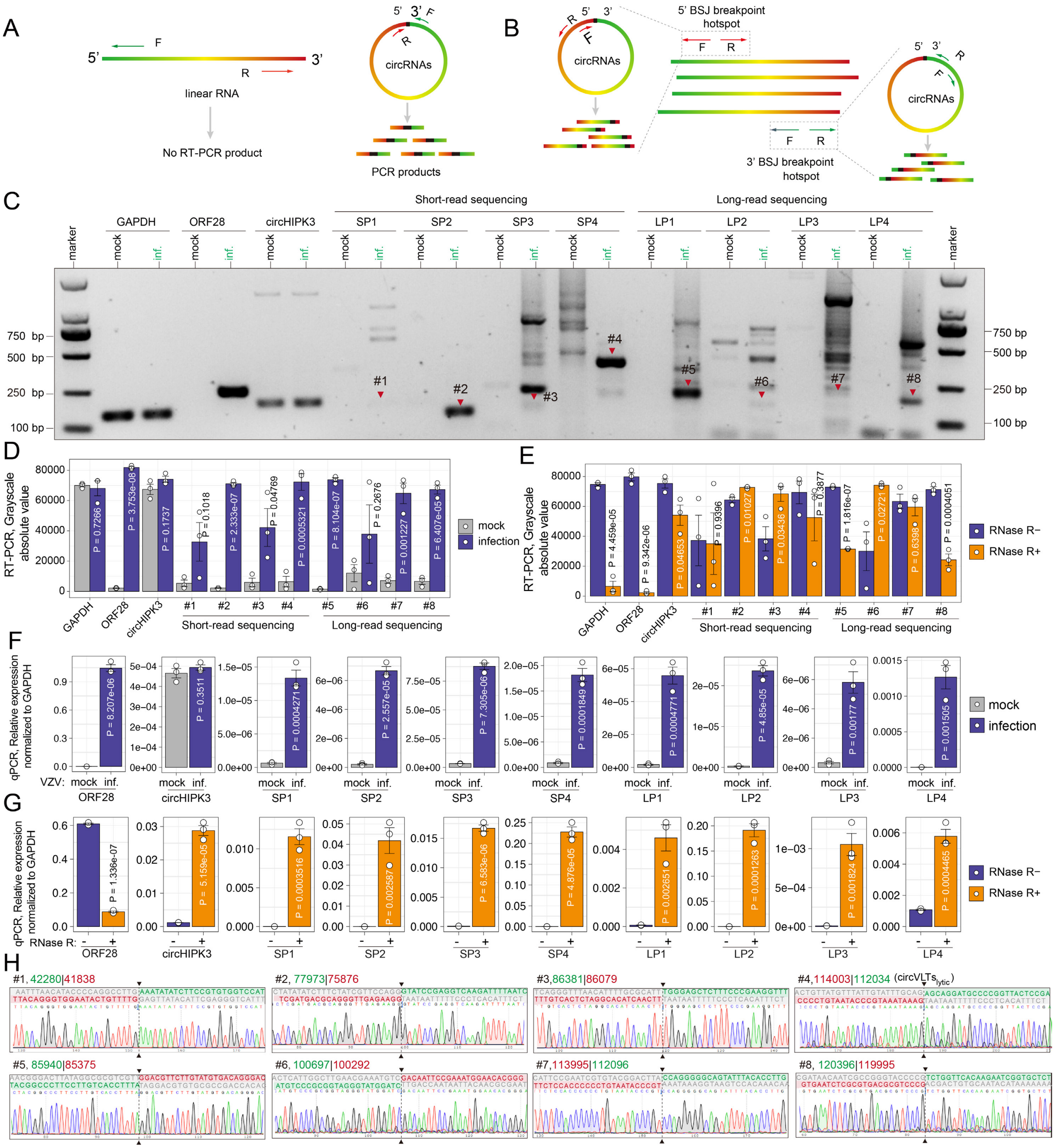

Supplementary Figure 5. Verification of VZV circRNAs.

(A) Illustration of how inverse RT-PCR with divergent primers selectively amplify different regions of circRNAs but not linear RNAs. **(B)** Schematic diagram of how divergent primers were designed to amplify predicted BSJs and hotspot regions of abundant circRNAs. **(C)** Inverse RT-PCR result of the VZV with primer sets shown in (A-B). Bands indicated by arrows were sequenced. **(D-E)** Relative expression of circRNAs of (C) and Figure 4B (E). **(F-G)** qPCR was

conducted to detect the expression of VZV circRNAs. N = 3 independent experiments were performed. Statistical comparisons between the VZV infection group and the mock group, or between the VZV infection group and the VZV infection with RNase R treatment group were performed using a two-tailed unpaired t test and the P value was shown. Data are presented as mean ± S.E.M. **(H)** Representative Sanger sequencing results. BSJ breakpoints are indicated by dashed lines. Donor (green), acceptor (red) sequences, and downstream/upstream sequences (grey) flanking the junction were aligned with the BSJ sequence. Source data are provided as a Source Data file.

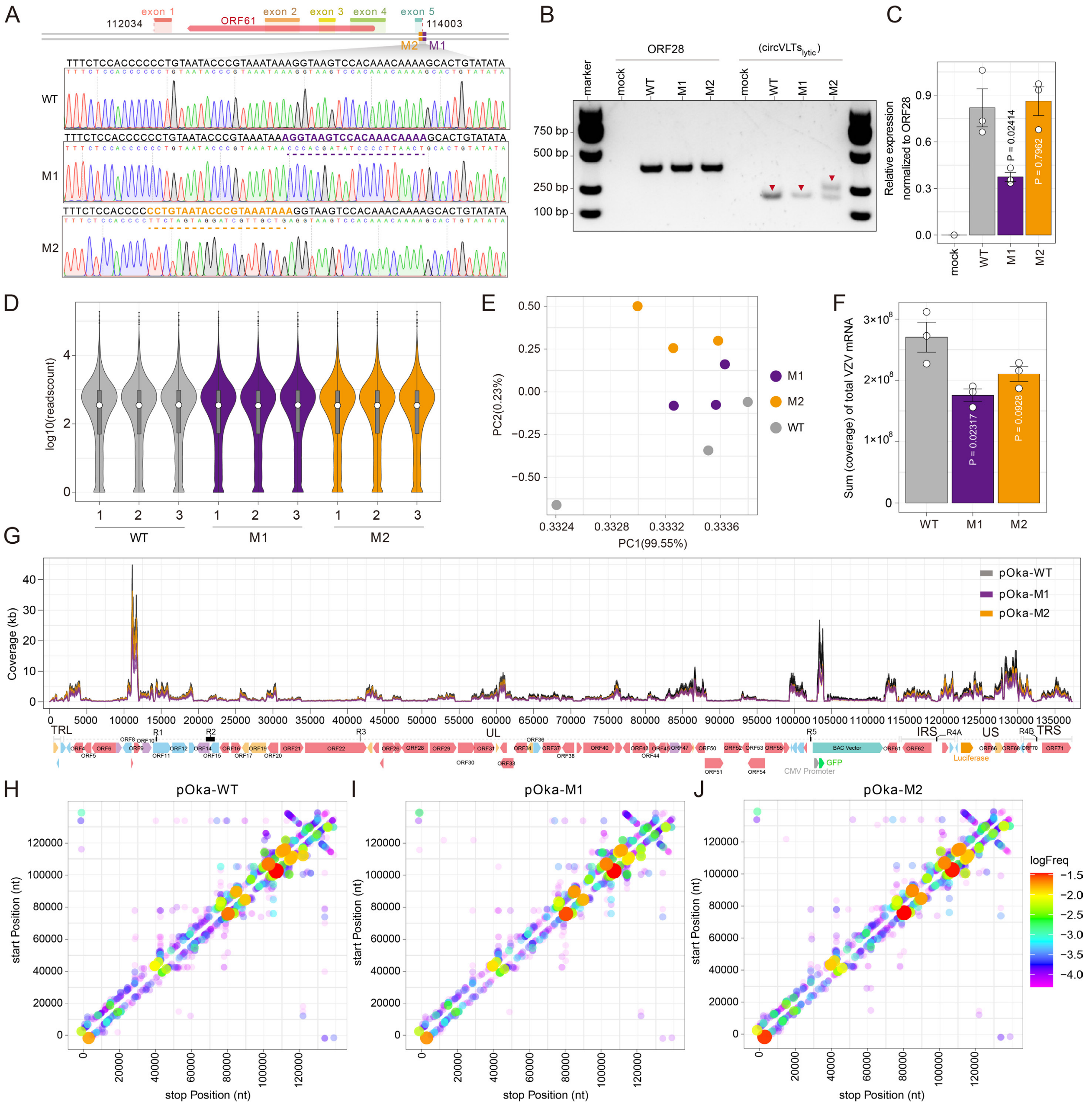

Supplementary Figure 6. Comparison of the transcriptional changes in the mutation of VZV circVLTs$_{lytic}$.

(A) Sanger sequencing results of the mutation downstream of circVLTs$_{lytic}$'s 5' splice donor in the genomic DNA position. **(B)** Inverse RT-PCR result of the VZV with primer sets of circVLTs$_{lytic}$ the relative expression was shown in **(C)**. **(D)** Gene expression distribution of each sample. **(E)** Principal component analysis (PCA) lot for the gene expression level of each sample. **(F)** Statistics of the total coverage of VZV RNA transcription. N = 3 independent experiments were performed.

Statistical comparisons were made with two-tailed unpaired t test. The P value of pOka-M1or pOka-M2 *vs.* pOka-WT group was shown. Data are presented as mean ± S.E.M. **(G)** Genome coverage of each sample. Genome organization of was shown in below. **(H-J)** ViReMa-based identification forward-splicing junction and back-splicing junction events in cells infected with pOka-WT (H), pOka-M1 (I), pOka-M2 (J). Source data are provided as a Source Data file.

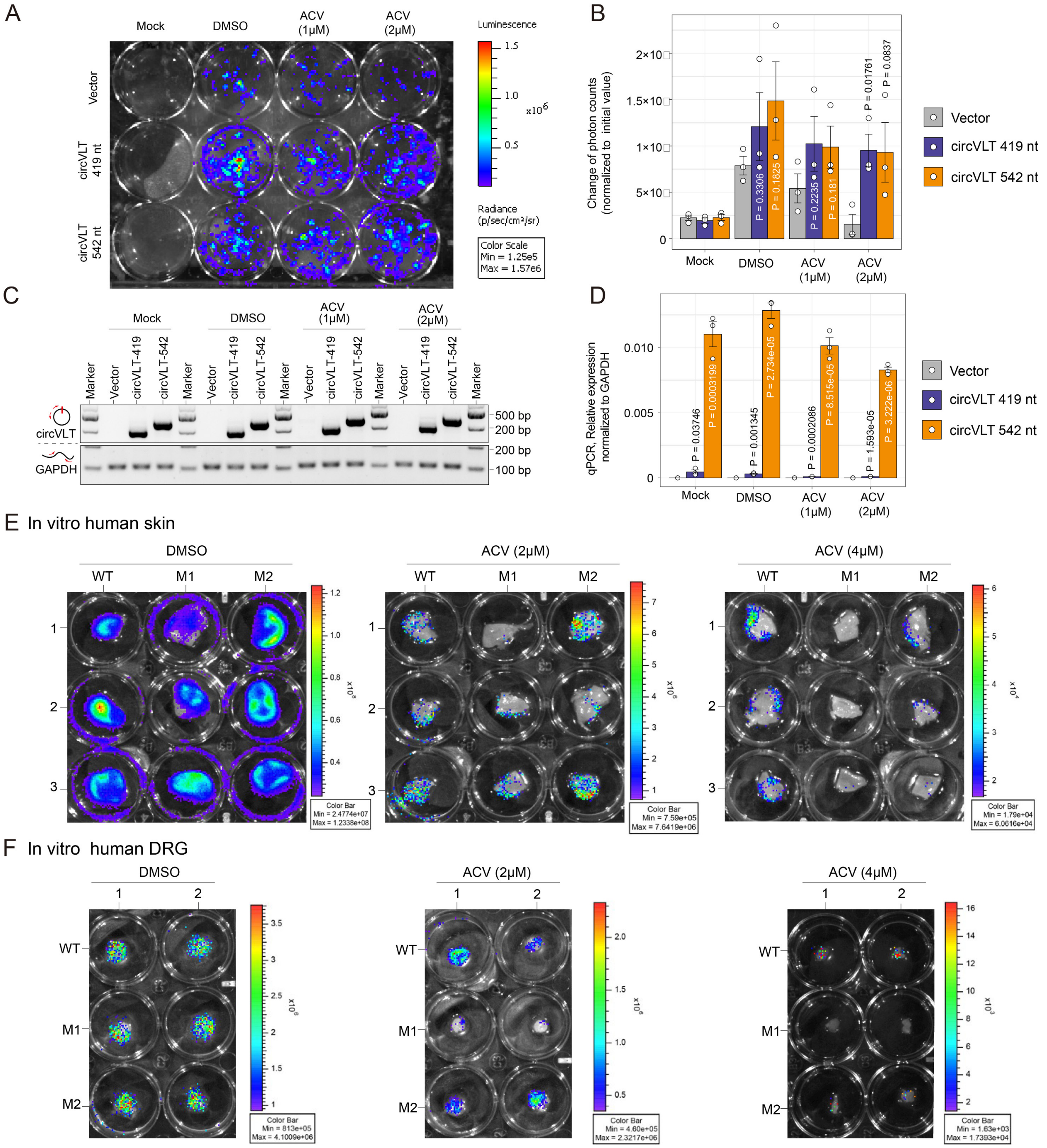

Supplementary Figure 7. The growth of pOka-WT, pOka-M1 and pOka-M2 in VZV infection models.

(A-B) Growth of pOka-M1 in SH-SY5Y cells, which were overexpressed with circVLT 419 nt or in circVLT 542 nt isoforms. The virus replication levels were measured by adding D-luciferin and recording the bioluminescence signal with an in vivo fluorescence imaging system (IVIS) system **(A)**. The average photon count values for each group were obtained from three independent experiments **(B)**. The relative photon counts were measured by removing the initial value before acyclovir treatment (day 0). **(C-D)** Inverse RT-PCR (C) or Inverse qPCR (D) were used to confirm the overexpression of circVLT 419 nt or in circVLT 542 nt isoforms. N = 3 independent experiments were performed. Statistical comparisons were conducted with a two-tailed unpaired t test. The P value of Vector *vs.* circVLT 419 nt or in circVLT 542 nt overexpression group was shown. Data are presented as mean ± S.E.M. **(E-F)** Growth of pOka-WT, pOka-M1 and pOka-M2 in vitro cultures of human skin (A) and human DRG (B). The virus replication levels were measured by adding D-luciferin and recording the bioluminescence signal with an IVIS system. Source data are provided as a Source Data file.

References

1 Jensen, N. J. *et al.* Analysis of the reiteration regions (R1 to R5) of varicella-zoster virus. *Virology* **546**, 38-50, doi:10.1016/j.virol.2020.03.008 (2020).

2 Zhang, Z. *et al.* Genome-wide mutagenesis reveals that ORF7 is a novel VZV skin-tropic factor. *PLoS Pathog* **6**, e1000971, doi:10.1371/journal.ppat.1000971 (2010).

3 Depledge, D. P. *et al.* A spliced latency-associated VZV transcript maps antisense to the viral transactivator gene 61. *Nat Commun* **9**, 1167, doi:10.1038/s41467-018-03569-2 (2018).
