# Supplementary material for: Identification and characterization of Varicella Zoster Virus circular RNA in lytic infection
Source: Nat Commun. 2024 Jun 10;15:4932. doi: 10.1038/s41467-024-49112-4 (PMC11164961; doi:10.1038/s41467-024-49112-4)
Supplement: Supplementary file 3 — Description of Additional Supplementary Files [file 41467_2024_49112_MOESM3_ESM.docx]

**Description of Additional Supplementary Files**

**Supplementary data 1. Human circRNAs identified by CIRI2 and find_circ.**

**Supplementary data 2. VZV circRNAs identified by CIRI2, find_circ and vircircRNA and reconstructed full-length circRNAs by CIRI-full.**

The localization of VZV circRNAs in reference genome of pOka-GFP-luciferase (PP054841) were also converted to pOka (AB097933.1) and vOka (KU926314.1) strain.

**Supplementary data 3. Experimentally confirmed VZV circRNAs, primers and probes used in this study.**

**Supplementary data 4. Sanger sequencing results of the mutation.**
