# Supplementary material for: Identification and characterization of Varicella Zoster Virus circular RNA in lytic infection
Source: Nat Commun. 2024 Jun 10;15:4932. doi: 10.1038/s41467-024-49112-4 (PMC11164961; doi:10.1038/s41467-024-49112-4)
Supplement: Supplementary file 6 — Supplementary data 3 [file 41467_2024_49112_MOESM6_ESM.docx]

| **Experimentally confirmed VZV circRNAs in VZV infected cells** | | | | | | | | |
| --- | --- | --- | --- | --- | --- | --- | --- | --- |
| **Clone**  **id** | **CircRNA id** | **pOka-GFP-luc**  **(PP054841)** | **vOka**  **(KU926314.1)** | **pOka**  **(AB097933.1)** | **Primer**  **name** | **Fragments**  **(AB097933.1)** | **Primer F**  **Sequence (5'to3')** | **Primer R**  **Sequence (5'to3')** |
| 1 | 1 | 41839\|42280 | 41839\|42280 | 42211\|41769 | SP1 | 41769-41880,42133-42211 | CCAGGCATGTCGGCGTATGAT | TTGGCGACGGCTGTCTTGTT |
| 2 | 2 | 41813\|42299 | 41813\|42299 | 42230\|41743 | SP1 | 41880-41743,42230-42133 | CCAGGCATGTCGGCGTATGAT | TTGGCGACGGCTGTCTTGTT |
| 3 | 2 | 41813\|42299 | 41813\|42299 | 42230\|41743 | SP1 | 41880-41743,42230-42133 | CCAGGCATGTCGGCGTATGAT | TTGGCGACGGCTGTCTTGTT |
| 4 | 3 | 41867\|42307 | 41867\|42307 | 42238\|41797 | SP1 | 41797-41880,42133-42238 | CCAGGCATGTCGGCGTATGAT | TTGGCGACGGCTGTCTTGTT |
| 5 | 4 | 41902\|42441 | 41902\|42442 | 42370\|41832 | SP1 | 41832-41880,42133-42370 | CCAGGCATGTCGGCGTATGAT | TTGGCGACGGCTGTCTTGTT |
| 6 | 5 | 41731\|42271 | 41731\|42271 | 42202\|41661 | SP1 | 41661-41880,42133-42202 | CCAGGCATGTCGGCGTATGAT | TTGGCGACGGCTGTCTTGTT |
| 7 | 6 | 41770\|42259 | 41770\|42259 | 42190\|41700 | SP1 | 41700-41880,42133-42190 | CCAGGCATGTCGGCGTATGAT | TTGGCGACGGCTGTCTTGTT |
| 8 | 7 | 41751\|42288 | 41751\|42288 | 42219\|41681 | SP1 | 41681-41880,42133-42219 | CCAGGCATGTCGGCGTATGAT | TTGGCGACGGCTGTCTTGTT |
| 9 | 8 | 41871\|42335 | 41871\|42335 | 42266\|41801 | SP1 | 41801-41880,42133-42266 | CCAGGCATGTCGGCGTATGAT | TTGGCGACGGCTGTCTTGTT |
| 10 | 9 | 41765\|42253 | 41765\|42253 | 42184\|41695 | SP1 | 41880-41695,42184-42134 | CCAGGCATGTCGGCGTATGAT | TTGGCGACGGCTGTCTTGTT |
| 11 | 10 | 75877\|77973 | 75879\|77975 | 77901\|75804 | SP2 | 75804-75854,77827-77901 | TGTACAGCATCAGGTCGAAT | TGACGACAGCGCTATATCCA |
| 12 | 10 | 75877\|77973 | 75879\|77975 | 77901\|75804 | SP2 | 75804-75854,77827-77901 | TGTACAGCATCAGGTCGAAT | TGACGACAGCGCTATATCCA |
| 13 | 11 | 86080\|86381 | 86082\|86383 | 86309\|86007 | SP3 | 86007-86084,86224-86309 | AACAGAGGACGCCGATAAATCC | GCAATGACCGCAATGCTGAT |
| 14 | 12 | 86101\|86355 | 86103\|86357 | 86283\|86028 | SP3 | 86028-86084,86222-86283 | AACAGAGGACGCCGATAAATCC | GCAATGACCGCAATGCTGAT |
| 15 | 13 | 85908\|86401 | 85910\|86403 | 86329\|85835 | SP3 | 85835-86084,86228-86329 | AACAGAGGACGCCGATAAATCC | GCAATGACCGCAATGCTGAT |
| 16 | 14 | 86074\|86420 | 86076\|86422 | 86348\|86001 | SP3 | 86291-86348,86001-86113 | AACAGAGGACGCCGATAAATCC | GCAATGACCGCAATGCTGAT |
| 17 | 15 | 86073\|86357 | 86075\|86359 | 86285\|86000 | SP3 | 86000-86084,86224-86285 | AACAGAGGACGCCGATAAATCC | GCAATGACCGCAATGCTGAT |
| 18 | 16 | 86016\|86405 | 86018\|86407 | 86333\|85943 | SP3 | 85943-86084,86215-86333 | AACAGAGGACGCCGATAAATCC | GCAATGACCGCAATGCTGAT |
| 19 | 17 | 85967\|86335 | 85969\|86337 | 86263\|85894 | SP3 | 85894-86084,86222-86263 | AACAGAGGACGCCGATAAATCC | GCAATGACCGCAATGCTGAT |
| 20 | 18 | 112034\|114003 | 102779\|104747 | 104785\|102816 | SP4 | 104406-104517,104731-104785,102816-102944,103787-103890 | ACGGTGCTCATGCATATGGT | AGTGCAATCCAACCTGCTGT |
| 21 | 19 | 85376\|85940 | 85378\|85942 | 85868\|85303 | LP1 | 85770-85868,85303-85372 | TACGGCTCTTCAAATTCCTGT | AATGTCGGTTTCTGCAGGGT |
| 22 | 20 | 85389\|85939 | 85391\|85941 | 85867\|85316 | LP1 | 85773-85867,85316-85372 | TACGGCTCTTCAAATTCCTGT | AATGTCGGTTTCTGCAGGGT |
| 23 | 21 | 85383\|85939 | 85385\|85941 | 85867\|85310 | LP1 | 85769-85867,85310-85371 | TACGGCTCTTCAAATTCCTGT | AATGTCGGTTTCTGCAGGGT |
| 24 | 22 | 100293\|100697 | 100295\|100699 | 100625\|100220 | LP2 | 100551-100625,100218-100311 | AATAACACCCCCTGCCTTGC | ACAGAAGACAACCCAACCGA |
| 25 | 23 | 112097\|113995 | 102841\|104739 | 104777\|102878 | LP3 | 104314-104394,104712-104777,102878-102939 | GAGAGGACACTGGACGCTG | TGTTGCTATTGGTCGTAAGGGT |
| 26 | 24 | 112063\|113574 | 102807\|104318 | 104356\|102844 | LP3 | 104317-104356,102844-102939 | GAGAGGACACTGGACGCTG | TGTTGCTATTGGTCGTAAGGGT |
| 27 | 25 | 112069\|113699 | 102813\|104443 | 104481\|102850 | LP3 | 104313-104481,102850-102939 | GAGAGGACACTGGACGCTG | TGTTGCTATTGGTCGTAAGGGT |
| 28 | 26 | 112163\|112956 | 102907\|103700 | 102944\|103738 | LP3-2 | 102829-102944,103787-103845 | GGAGGTCCATGCCCGAATAC | TGAATCTGCACAGCAACCCC |
| 29 | 27 | 119996\|120396 | 110732\|111132 | 111311\|110910 | LP4 | 110982-110910,111311-111239 | ATTCCTCCCAGCACGCTAAC | CATCGGACGGGGAAGACTTT |
| 30 | 28 | 117533\|117967 | 108277\|108711 | 108749\|108314 | LP5 | 108427-108314,108749-108595 | GTAAAAAGCCGGGCGATGAG | GGGGGATACAGGGGAGAAGG |
| 31 | 29 | 443\|947 | 443\|947 | 947\|442 | SP5 | 442-610,767-947 | TAAGAACCGCCGGTGGTCTT | CGACAAGCTGCAAGCGAGAA |
| 32 | 30 | 324\|856 | 324\|856 | 856\|323 | SP5 | 323-610,767-856 | TAAGAACCGCCGGTGGTCTT | CGACAAGCTGCAAGCGAGAA |
| 33 | 31 | 256\|895 | 256\|895 | 895\|255 | SP5 | 255-610,767-895 | TAAGAACCGCCGGTGGTCTT | CGACAAGCTGCAAGCGAGAA |
| 34 | 32 | 476\|959 | 476\|959 | 959\|475 | SP5 | 475-610,767-959 | TAAGAACCGCCGGTGGTCTT | CGACAAGCTGCAAGCGAGAA |
| 35 | 33 | 473\|959 | 473\|959 | 959\|472 | SP5 | 472-610,767-959 | TAAGAACCGCCGGTGGTCTT | CGACAAGCTGCAAGCGAGAA |
| 36 | 34 | 312\|851 | 312\|851 | 851\|311 | SP5 | 311-609,764-851 | TAAGAACCGCCGGTGGTCTT | CGACAAGCTGCAAGCGAGAA |
| 37 | 34 | 312\|851 | 312\|851 | 851\|311 | SP5 | 311-610,764-851 | TAAGAACCGCCGGTGGTCTT | CGACAAGCTGCAAGCGAGAA |
| 38 | 35 | 322\|902 | 322\|902 | 902\|321 | SP5 | 321-610,767-902 | TAAGAACCGCCGGTGGTCTT | CGACAAGCTGCAAGCGAGAA |
| 39 | 36 | 419\|851 | 419\|851 | 851\|418 | SP5 | 418-610,767-851 | TAAGAACCGCCGGTGGTCTT | CGACAAGCTGCAAGCGAGAA |
| 40 | 37 | 313\|869 | 313\|869 | 869\|312 | SP5 | 312-610,767-869 | TAAGAACCGCCGGTGGTCTT | CGACAAGCTGCAAGCGAGAA |
| 41 | 38 | 455\|917 | 455\|917 | 917\|454 | SP5 | 454-610,767-917 | TAAGAACCGCCGGTGGTCTT | CGACAAGCTGCAAGCGAGAA |
| 42 | 39 | 3184\|4091 | 3184\|4091 | 4091\|3183 | SP6-1 | 3183-3398,3975-4091 | TAGACGTTGTGGAGTCCTCG | CGGAGTTGATGTTGTACGGACA |
| 43 | 40 | 3179\|4044 | 3179\|4044 | 4044\|3178 | SP6-1 | 3178-3398,3975-4044 | TAGACGTTGTGGAGTCCTCG | CGGAGTTGATGTTGTACGGACA |
| 44 | 41 | 3181\|4049 | 3181\|4049 | 4049\|3180 | SP6-1 | 3180-3398,3975-4049 | TAGACGTTGTGGAGTCCTCG | CGGAGTTGATGTTGTACGGACA |
| 45 | 42 | 3199\|4046 | 3199\|4046 | 4046\|3198 | SP6-1 | 3398-3198,4046-3975 | TAGACGTTGTGGAGTCCTCG | CGGAGTTGATGTTGTACGGACA |
| 46 | 43 | 3297\|4049 | 3297\|4049 | 4049\|3296 | SP6-1 | 3975-4049,3296-3398 | TAGACGTTGTGGAGTCCTCG | CGGAGTTGATGTTGTACGGACA |
| 47 | 44 | 3239\|4086 | 3239\|4086 | 4086\|3238 | SP6-1 | 3238-3398,3975-4086 | TAGACGTTGTGGAGTCCTCG | CGGAGTTGATGTTGTACGGACA |
| 48 | 45 | 3174\|4044 | 3174\|4044 | 4044\|3173 | SP6-1 | 3173-3395,3975-4044 | TAGACGTTGTGGAGTCCTCG | CGGAGTTGATGTTGTACGGACA |
| 49 | 46 | 3523\|4046 | 3523\|4046 | 4046\|3522 | SP6-1 | 3522-3795,3975-4046 | TAGACGTTGTGGAGTCCTCG | CGGAGTTGATGTTGTACGGACA |
| 50 | 47 | 3669\|4083 | 3669\|4083 | 4083\|3668 | SP6-2 | 3668-3795,3975-4083 | TAGACGTTGTGGAGTCCTCG | GGACATCGACGATCATCCAGA |
| 51 | 48 | 3468\|4004 | 3468\|4004 | 4004\|3467 | SP6-2 | 3467-3795,3975-4004 | TAGACGTTGTGGAGTCCTCG | GGACATCGACGATCATCCAGA |
| 52 | 49 | 3673\|4010 | 3673\|4010 | 4010\|3672 | SP6-2 | 3672-3795,3975-4010 | TAGACGTTGTGGAGTCCTCG | GGACATCGACGATCATCCAGA |
| 53 | 50 | 3694\|4174 | 3694\|4174 | 4174\|3693 | SP6-2 | 3693-3795,3975-4174 | TAGACGTTGTGGAGTCCTCG | GGACATCGACGATCATCCAGA |
| 54 | 51 | 3666\|4112 | 3666\|4112 | 4112\|3665 | SP6-2 | 3975-4112,3665-3795 | TAGACGTTGTGGAGTCCTCG | GGACATCGACGATCATCCAGA |
| 55 | 52 | 3718\|4092 | 3718\|4092 | 4092\|3717 | SP6-2 | 3717-3795,3975-4092 | TAGACGTTGTGGAGTCCTCG | GGACATCGACGATCATCCAGA |
| 56 | 52 | 3718\|4092 | 3718\|4092 | 4092\|3717 | SP6-2 | 3717-3795,3975-4092 | TAGACGTTGTGGAGTCCTCG | GGACATCGACGATCATCCAGA |
| 57 | 52 | 3718\|4092 | 3718\|4092 | 4092\|3717 | SP6-2 | 3717-3795,3975-4092 | TAGACGTTGTGGAGTCCTCG | GGACATCGACGATCATCCAGA |
| 58 | 53 | 3704\|4042 | 3704\|4042 | 4042\|3703 | SP6-2 | 3703-3795,3975-4042 | TAGACGTTGTGGAGTCCTCG | GGACATCGACGATCATCCAGA |
| 59 | 54 | 3692\|4048 | 3692\|4048 | 4048\|3691 | SP6-2 | 3691-3795,3975-4048 | TAGACGTTGTGGAGTCCTCG | GGACATCGACGATCATCCAGA |
| 60 | 55 | 3370\|4150 | 3370\|4150 | 4150\|3369 | SP7 | 3369-3570,4054-4150 | ATCCGAAGGTTCGTCTGGTAGC | GGCCGTGCTATTGAAGTCGTC |
| 61 | 56 | 3497\|4260 | 3497\|4260 | 4260\|3496 | SP7 | 3496-3570,4054-4260 | ATCCGAAGGTTCGTCTGGTAGC | GGCCGTGCTATTGAAGTCGTC |
| 62 | 57 | 3471\|4192 | 3471\|4192 | 4192\|3470 | SP7 | 3470-3570,4058-4192 | ATCCGAAGGTTCGTCTGGTAGC | GGCCGTGCTATTGAAGTCGTC |
| 63 | 58 | 3432\|4146 | 3432\|4146 | 4146\|3431 | SP7 | 3431-3570,4054-4146 | ATCCGAAGGTTCGTCTGGTAGC | GGCCGTGCTATTGAAGTCGTC |
| 64 | 59 | 3502\|4152 | 3502\|4152 | 4152\|3501 | SP7 | 3501-3570,4054-4152 | ATCCGAAGGTTCGTCTGGTAGC | GGCCGTGCTATTGAAGTCGTC |
| 65 | 60 | 3252\|4109 | 3252\|4109 | 4109\|3251 | SP7 | 3251-3570,4054-4109 | ATCCGAAGGTTCGTCTGGTAGC | GGCCGTGCTATTGAAGTCGTC |
| 66 | 61 | 3333\|4084 | 3333\|4084 | 4084\|3332 | SP7 | 3332-3570,4054-4084 | ATCCGAAGGTTCGTCTGGTAGC | GGCCGTGCTATTGAAGTCGTC |
| 67 | 62 | 3283\|4093 | 3283\|4093 | 4093\|3282 | SP7 | 3282-3570,4054-4093 | ATCCGAAGGTTCGTCTGGTAGC | GGCCGTGCTATTGAAGTCGTC |
| 68 | 63 | 3264\|4191 | 3264\|4191 | 4191\|3263 | SP7 | 3263-3570,4054-4191 | ATCCGAAGGTTCGTCTGGTAGC | GGCCGTGCTATTGAAGTCGTC |
| 69 | 64 | 3428\|4154 | 3428\|4154 | 4154\|3427 | SP7 | 3427-3570,4054-4154 | ATCCGAAGGTTCGTCTGGTAGC | GGCCGTGCTATTGAAGTCGTC |
| 70 | 65 | 3499\|4364 | 3499\|4364 | 4364\|3498 | SP7 | 3498-3570,4054-4364 | ATCCGAAGGTTCGTCTGGTAGC | GGCCGTGCTATTGAAGTCGTC |
| 71 | 66 | 3506\|4152 | 3506\|4152 | 4152\|3505 | SP7 | 3505-3570,4054-4152 | ATCCGAAGGTTCGTCTGGTAGC | GGCCGTGCTATTGAAGTCGTC |
| 72 | 67 | 11217\|11761 | 11217\|11761 | 11761\|11216 | SP8 | 11718-11761,11216-11435 | AGCAGACCTAGGTGAAGGAGCATC | GCTCGCTTCGGTGAATCCTC |
| 73 | 68 | 11126\|11779 | 11126\|11779 | 11779\|11125 | SP8 | 11125-11435,11718-11779 | AGCAGACCTAGGTGAAGGAGCATC | GCTCGCTTCGGTGAATCCTC |
| 74 | 69 | 11115\|11806 | 11115\|11806 | 11806\|11114 | SP8 | 11114-11435,11718-11806 | AGCAGACCTAGGTGAAGGAGCATC | GCTCGCTTCGGTGAATCCTC |
| 75 | 70 | 11129\|11779 | 11129\|11779 | 11779\|11128 | SP8 | 11128-11435,11718-11779 | AGCAGACCTAGGTGAAGGAGCATC | GCTCGCTTCGGTGAATCCTC |
| 76 | 71 | 11358\|11921 | 11358\|11921 | 11921\|11357 | SP8 | 11357-11435,11718-11921 | AGCAGACCTAGGTGAAGGAGCATC | GCTCGCTTCGGTGAATCCTC |
| 77 | 72 | 11150\|11761 | 11150\|11761 | 11761\|11149 | SP8 | 11357-11435,11718-11921 | AGCAGACCTAGGTGAAGGAGCATC | GCTCGCTTCGGTGAATCCTC |
| 78 | 73 | 11206\|11767 | 11206\|11767 | 11767\|11205 | SP8 | 11205-11435,11718-11767 | AGCAGACCTAGGTGAAGGAGCATC | GCTCGCTTCGGTGAATCCTC |
| 79 | 75 | 11401\|13060 | 11401\|13060 | 13060\|11400 | SP8 | 11400-11433,12663-13060 | AGCAGACCTAGGTGAAGGAGCATC | GCTCGCTTCGGTGAATCCTC |
| 80 | 76 | 49756\|52061 | 49757\|52062 | 51990\|49684 | SP9 | 49684-50243,51923-51990 | GATGACCGAAGCCAAGGATG | CTGGCCTAGACGCGTGAATA |
| 81 | 77 | 49968\|52169 | 49969\|52170 | 52098\|49896 | SP9 | 49896-50243,51924-52098 | GATGACCGAAGCCAAGGATG | CTGGCCTAGACGCGTGAATA |
| 82 | 78 | 50089\|52094 | 50090\|52095 | 52023\|50017 | SP9 | 50017-50243,52023-51923 | GATGACCGAAGCCAAGGATG | CTGGCCTAGACGCGTGAATA |
| 83 | 79 | 49979\|52268 | 49980\|52269 | 52197\|49907 | SP9 | 49907-50243,51923-52197 | GATGACCGAAGCCAAGGATG | CTGGCCTAGACGCGTGAATA |
| 84 | 80 | 50236\|52516 | 50237\|52517 | 52445\|50164 | SP9 | 50164-50243,51923-52106,52300-52445 | GATGACCGAAGCCAAGGATG | CTGGCCTAGACGCGTGAATA |
| 85 | 81 | 50063\|52175 | 50064\|52176 | 52104\|49991 | SP9 | 49991-50243,51923-52104 | GATGACCGAAGCCAAGGATG | CTGGCCTAGACGCGTGAATA |
| 86 | 82 | 49993\|52260 | 49994\|52261 | 52189\|49921 | SP9 | 49921-50243,51923-52189 | GATGACCGAAGCCAAGGATG | CTGGCCTAGACGCGTGAATA |
| 87 | 83 | 54178\|54744 | 54179\|54745 | 54673\|54106 | SP10 | 54106-54284,54628-54673 | ATCGGTTAGCGGCTAACTGT | ATCAAAGAGTTCGTCATCTTCGG |
| 88 | 84 | 54221\|54793 | 54222\|54794 | 54722\|54149 | SP10 | 54149-54284,54628-54722 | ATCGGTTAGCGGCTAACTGT | ATCAAAGAGTTCGTCATCTTCGG |
| 89 | 84 | 54221\|54793 | 54222\|54794 | 54722\|54149 | SP10 | 54149-54284,54628-54722 | ATCGGTTAGCGGCTAACTGT | ATCAAAGAGTTCGTCATCTTCGG |
| 90 | 84 | 54221\|54793 | 54222\|54794 | 54722\|54149 | SP10 | 54149-54284,54628-54722 | ATCGGTTAGCGGCTAACTGT | ATCAAAGAGTTCGTCATCTTCGG |
| 91 | 85 | 54291\|54737 | 54292\|54738 | 54666\|54219 | SP10 | 54219-54284,54628-54666 | ATCGGTTAGCGGCTAACTGT | ATCAAAGAGTTCGTCATCTTCGG |
| 92 | 86 | 54308\|54775 | 54309\|54776 | 54704\|54236 | SP10 | 54628-54704,54236-54284 | ATCGGTTAGCGGCTAACTGT | ATCAAAGAGTTCGTCATCTTCGG |
| 93 | 87 | 54294\|54821 | 54295\|54822 | 54750\|54222 | SP10 | 54628-54750,54222-54284 | ATCGGTTAGCGGCTAACTGT | ATCAAAGAGTTCGTCATCTTCGG |
| 94 | 88 | 55775\|56245 | 55776\|56246 | 56174\|55703 | SP11 | 56061-56174,55703-55783 | GGAGAGGCCTTATTGCAGAC | CGGCTTCGTTATCATGACTCG |
| 95 | 89 | 55711\|56168 | 55712\|56169 | 56097\|55639 | SP11 | 55639-55783,56061-56097 | GGAGAGGCCTTATTGCAGAC | CGGCTTCGTTATCATGACTCG |
| 96 | 89 | 55711\|56168 | 55712\|56169 | 56097\|55639 | SP11 | 55639-55783,56061-56097 | GGAGAGGCCTTATTGCAGAC | CGGCTTCGTTATCATGACTCG |
| 97 | 90 | 55587\|56179 | 55588\|56180 | 56108\|55515 | SP11 | 55515-55783,56061-56108 | GGAGAGGCCTTATTGCAGAC | CGGCTTCGTTATCATGACTCG |
| 98 | 91 | 55737\|56237 | 55738\|56238 | 56166\|55665 | SP11 | 55665-55783,56061-56166 | GGAGAGGCCTTATTGCAGAC | CGGCTTCGTTATCATGACTCG |
| 99 | 92 | 55816\|56306 | 55817\|56307 | 56235\|55744 | SP11 | 56061-56235,55744-55783 | GGAGAGGCCTTATTGCAGAC | CGGCTTCGTTATCATGACTCG |
| 100 | 93 | 66362\|67012 | 66364\|67014 | 66940\|66289 | SP12 | 66289-66337,66896-66940 | ATGCTACGGTCGATATATGCG | TGTCAAAGAAGCTCACGTCG |
| 101 | 94 | 66348\|67214 | 66350\|67216 | 67142\|66275 | SP12 | 66275-66337,66896-67142 | ATGCTACGGTCGATATATGCG | TGTCAAAGAAGCTCACGTCG |
| 102 | 95 | 66310\|67035 | 66312\|67037 | 66963\|66237 | SP12 | 66237-66337,66896-66963 | ATGCTACGGTCGATATATGCG | TGTCAAAGAAGCTCACGTCG |
| 103 | 96 | 77608\|77973 | 77610\|77975 | 77901\|77535 | SP13 | 77535-77653,77827-77901 | TGTACAGCATCAGGTCGAAT | GATGTCACAGCTTGTTCGTG |
| 104 | 97 | 77612\|77971 | 77614\|77973 | 77899\|77539 | SP13 | 77539-77653,77821-77899 | TGTACAGCATCAGGTCGAAT | GATGTCACAGCTTGTTCGTG |
| 105 | 98 | 77638\|79673 | 77640\|79675 | 79601\|77565 | SP13 | 77565-77821,77827-77902,79532-79601 | TGTACAGCATCAGGTCGAAT | GATGTCACAGCTTGTTCGTG |
| 106 | 99 | 78139\|78966 | 78141\|78968 | 78894\|78066 | SP14 | 78066-78118,78579-78894 | TACGCAGAGCATATGGTGGC | GCGTTAGGTATCACAACGTGTAGTA |
| 107 | 100 | 77732\|78913 | 77734\|78915 | 78841\|77659 | SP14 | 77659-77730,78004-78115,78579-78841 | TACGCAGAGCATATGGTGGC | GCGTTAGGTATCACAACGTGTAGTA |
| 108 | 101 | 75446\|79139 | 75448\|79141 | 79067\|75373 | SP14 | 75373-75854,78768-79067 | TACGCAGAGCATATGGTGGC | GCGTTAGGTATCACAACGTGTAGTA |
| 109 | 102 | 78077\|78776 | 78079\|78778 | 78704\|78004 | SP14 | 78004-78118,78579-78704 | TACGCAGAGCATATGGTGGC | GCGTTAGGTATCACAACGTGTAGTA |
| 110 | 103 | 75749\|76175 | 75751\|76177 | 76103\|75676 | SP15 | 75676-75854,76025-76102 | GTAATGGCGATTACATCTGC | TGACGACAGCGCTATATCCA |
| 111 | 104 | 75565\|76209 | 75567\|76211 | 76137\|75492 | SP15 | 75492-75854,76025-76137 | GTAATGGCGATTACATCTGC | TGACGACAGCGCTATATCCA |
| 112 | 105 | 75751\|76168 | 75753\|76170 | 76096\|75678 | SP15 | 75678-75854,76025-76096 | GTAATGGCGATTACATCTGC | TGACGACAGCGCTATATCCA |
| 113 | 106 | 75835\|76346 | 75837\|76348 | 76274\|75762 | SP15 | 76025-76274,75762-75854 | GTAATGGCGATTACATCTGC | TGACGACAGCGCTATATCCA |
| 114 | 107 | 75833\|76346 | 75835\|76348 | 76274\|75760 | SP15 | 75760-75854,76025-76274 | GTAATGGCGATTACATCTGC | TGACGACAGCGCTATATCCA |
| 115 | 108 | 75896\|76471 | 75898\|76473 | 76399\|75823 | SP15 | 76025-76399,75823-75854 | GTAATGGCGATTACATCTGC | TGACGACAGCGCTATATCCA |
| 116 | 109 | 75683\|76204 | 75685\|76206 | 76132\|75610 | SP15 | 75610-75854,76025-76132 | GTAATGGCGATTACATCTGC | TGACGACAGCGCTATATCCA |
| 117 | 110 | 75649\|79238 | 75651\|79240 | 79166\|75576 | SP16-1 | 75576-75854,78768-79166 | AACATTGTTAACTCCAGGCACC | TGACGACAGCGCTATATCCA |
| 118 | 111 | 75477\|78880 | 75479\|78882 | 78808\|75404 | SP16-1 | 75404-75854,78768-78808 | AACATTGTTAACTCCAGGCACC | TGACGACAGCGCTATATCCA |
| 119 | 112 | 75639\|79247 | 75641\|79249 | 79175\|75566 | SP16-1 | 75566-758547,78768-79175 | AACATTGTTAACTCCAGGCACC | TGACGACAGCGCTATATCCA |
| 120 | 113 | 75886\|79308 | 75888\|79310 | 79236\|75813 | SP16-1 | 75813-75854,78770-79236 | AACATTGTTAACTCCAGGCACC | TGACGACAGCGCTATATCCA |
| 121 | 114 | 75607\|78919 | 75609\|78921 | 78847\|75534 | SP16-1 | 75534-75854,78768-78847 | AACATTGTTAACTCCAGGCACC | TGACGACAGCGCTATATCCA |
| 122 | 115 | 75764\|78964 | 75766\|78966 | 78892\|75691 | SP16-1 | 75691-75854,78768-78892 | AACATTGTTAACTCCAGGCACC | TGACGACAGCGCTATATCCA |
| 123 | 115 | 75764\|78964 | 75766\|78966 | 78892\|75691 | SP16-1 | 75691-75854,78768-78892 | AACATTGTTAACTCCAGGCACC | TGACGACAGCGCTATATCCA |
| 124 | 116 | 75767\|78935 | 75769\|78937 | 78863\|75694 | SP16-1 | 75694-75854,78768-78863 | AACATTGTTAACTCCAGGCACC | TGACGACAGCGCTATATCCA |
| 125 | 117 | 75595\|78880 | 75597\|78882 | 78808\|75522 | SP16-1 | 75522-75854,78768-78808 | AACATTGTTAACTCCAGGCACC | TGACGACAGCGCTATATCCA |
| 126 | 117 | 75595\|78880 | 75597\|78882 | 78808\|75522 | SP16-1 | 75522-75854,78768-78808 | AACATTGTTAACTCCAGGCACC | TGACGACAGCGCTATATCCA |
| 127 | 118 | 80761\|81665 | 80763\|81667 | 81593\|80688 | SP16-1 | 80688-81141,81509-81593 | AACATTGTTAACTCCAGGCACC | TGACGACAGCGCTATATCCA |
| 128 | 119 | 75730\|83598 | 75732\|83600 | 83526\|75657 | SP16-2 | 75657-75854,83099-83526 | TACCGACGGACATGAACCAA | TGACGACAGCGCTATATCCA |
| 129 | 120 | 75442\|83315 | 75444\|83317 | 83243\|75369 | SP16-2 | 75369-75854,83099-83243 | TACCGACGGACATGAACCAA | TGACGACAGCGCTATATCCA |
| 130 | 121 | 75770\|83359 | 75772\|83361 | 83287\|75697 | SP16-2 | 75697-75854,83099-83287 | TACCGACGGACATGAACCAA | TGACGACAGCGCTATATCCA |
| 131 | 122 | 75876\|83276 | 75878\|83278 | 83204\|75803 | SP16-2 | 75803-75854,83099-83204 | TACCGACGGACATGAACCAA | TGACGACAGCGCTATATCCA |
| 132 | 123 | 75702\|83356 | 75704\|83358 | 83284\|75629 | SP16-2 | 75629-75854,83099-83284 | TACCGACGGACATGAACCAA | TGACGACAGCGCTATATCCA |
| 133 | 124 | 81928\|83364 | 81930\|83366 | 83292\|81855 | SP16-3 | 81855-82206,83099-83292 | TACCGACGGACATGAACCAA | TGTTCTCGGCTACTGCTCGA |
| 134 | 125 | 82174\|83344 | 82176\|83346 | 83272\|82101 | SP16-3 | 82101-82206,83099-83272 | TACCGACGGACATGAACCAA | TGTTCTCGGCTACTGCTCGA |
| 135 | 126 | 82054\|83393 | 82056\|83395 | 83321\|81981 | SP16-3 | 81981-82206,83099-83321 | TACCGACGGACATGAACCAA | TGTTCTCGGCTACTGCTCGA |
| 136 | 127 | 82224\|83438 | 82226\|83440 | 83366\|82151 | SP16-3 | 82151-82206,83099-83366 | TACCGACGGACATGAACCAA | TGTTCTCGGCTACTGCTCGA |
| 137 | 128 | 81138\|83351 | 81140\|83353 | 83279\|81065 | SP16-3 | 81065-81440,83099-83279 | TACCGACGGACATGAACCAA | TGTTCTCGGCTACTGCTCGA |
| 138 | 129 | 77606\|83280 | 77608\|83282 | 83208\|77533 | SP16-4 | 77533-77899,81397-81440,83099-83208 | TACCGACGGACATGAACCAA | GTCTTACAGCACAGCGGTGT |
| 139 | 130 | 81131\|83326 | 81133\|83328 | 83254\|81058 | SP16-4 | 81058-81440,83099-83254 | TACCGACGGACATGAACCAA | GTCTTACAGCACAGCGGTGT |
| 140 | 131 | 81424\|83283 | 81426\|83285 | 83211\|81351 | SP16-4 | 81351-81440,83099-83211 | TACCGACGGACATGAACCAA | GTCTTACAGCACAGCGGTGT |
| 141 | 132 | 81467\|83274 | 81469\|83276 | 83202\|81394 | SP16-4 | 81394-81440,83099-83202 | TACCGACGGACATGAACCAA | GTCTTACAGCACAGCGGTGT |
| 142 | 133 | 81441\|83333 | 81443\|83335 | 83261\|81368 | SP16-4 | 81368-81440,83099-83261 | TACCGACGGACATGAACCAA | GTCTTACAGCACAGCGGTGT |
| 143 | 134 | 81297\|83325 | 81299\|83327 | 83253\|81224 | SP16-4 | 81224-81440,83099-83253 | TACCGACGGACATGAACCAA | GTCTTACAGCACAGCGGTGT |
| 144 | 135 | 80858\|81793 | 80860\|81795 | 81721\|80785 | SP17 | 80785-81141,81509-81721 | TCCAGGCGAGACTTGATACC | CCGCTATAACGTCGCCAAGA |
| 145 | 136 | 85376\|85948 | 85378\|85950 | 85876\|85303 | SP18 | 85303-85354,85816-85876 | TCCGGTTACTTTCCAGCGCTA | GTGGGGATTTAGGGTGCCAGTT |
| 146 | 137 | 85389\|85948 | 85391\|85950 | 85876\|85316 | SP18 | 85316-85354,85816-85867 | TCCGGTTACTTTCCAGCGCTA | GTGGGGATTTAGGGTGCCAGTT |
| 147 | 138 | 85383\|85948 | 85385\|85950 | 85876\|85310 | SP18 | 85310-85354,85816-85867 | TCCGGTTACTTTCCAGCGCTA | GTGGGGATTTAGGGTGCCAGTT |
| 148 | 139 | 85378\|85938 | 85380\|85940 | 85866\|85305 | SP18 | 85305-85354,85816-85866 | TCCGGTTACTTTCCAGCGCTA | GTGGGGATTTAGGGTGCCAGTT |
| 149 | 140 | 85366\|85988 | 85368\|85990 | 85916\|85293 | SP18 | 85293-85354,85816-85916 | TCCGGTTACTTTCCAGCGCTA | GTGGGGATTTAGGGTGCCAGTT |
| 150 | 141 | 85389\|85929 | 85391\|85931 | 85857\|85316 | SP18 | 85316-85354,85816-85857 | TCCGGTTACTTTCCAGCGCTA | GTGGGGATTTAGGGTGCCAGTT |
| 151 | 142 | 93594\|95374 | 93596\|95376 | 95302\|93521 | SP19 | 93521-93558,94722-94798,95224-95302 | CCGTGTTGTATCGTTCTCGCC | GGTTGGATGTGAGCGACGTGA |
| 152 | 143 | 93469\|94988 | 93471\|94990 | 94916\|93396 | SP19 | 93396-93558,94722-94916 | CCGTGTTGTATCGTTCTCGCC | GGTTGGATGTGAGCGACGTGA |
| 153 | 144 | 93556\|94894 | 93558\|94896 | 94822\|93483 | SP19 | 93483-93558,94722-94882 | CCGTGTTGTATCGTTCTCGCC | GGTTGGATGTGAGCGACGTGA |
| 154 | 145 | 111781\|113068 | 101944\|103812 | 103850\|101010 | SP20-1 | 101010-101138,102816-102881,103786-103850 | ACAGATACTGGACGATCACG | CTGGTAAGTCCGTACACGAT |
| 155 | 146 | 111781\|113040 | 101944\|103784 | 103822\|101010 | SP20-1 | 101010-101191,102814-102881,103786-103822 | ACAGATACTGGACGATCACG | CTGGTAAGTCCGTACACGAT |
| 156 | 151 | 111784\|113108 | 102526\|103852 | 103890\|102563 | SP20-1 | 102563-102881,103786-103890 | ACAGATACTGGACGATCACG | CTGGTAAGTCCGTACACGAT |
| 157 | 152 | 100013\|102141 | 101944\|104350 | 104388\|99940 | SP20-1 | 99940-100123,101009-101138,102816-102881,103786-103890,104252-104388, | ACAGATACTGGACGATCACG | CTGGTAAGTCCGTACACGAT |
| 158 | 18 | 112034\|114003 | 102779\|104747 | 104785\|102816 | SP20-1 | 102816-102881,103786-103890,104252-104394,104729-104785 | ACAGATACTGGACGATCACG | CTGGTAAGTCCGTACACGAT |
| 159 | 153 | 112045\|119093 | 102789\|109837 | 110726\|102826 | SP20-1 | 102826-102881,110617-110726,103786-103890,104252-104394,104729-104786 | ACAGATACTGGACGATCACG | CTGGTAAGTCCGTACACGAT |
| 160 | 154 | 112055\|113082 | 102799\|103826 | 103864\|102836 | SP20-2 | 102836-103220,103786-103864 | ACAGATACTGGACGATCACG | CTGAGTCTGATTCGGCATGT |
| 161 | 155 | 112063\|113051 | 102807\|103795 | 103833\|102844 | SP20-2 | 102844-103220,103786-103833 | ACAGATACTGGACGATCACG | CTGAGTCTGATTCGGCATGT |
| 162 | 156 | 112243\|113037 | 102987\|103781 | 103819\|103024 | SP20-2 | 103024-103220,103786-103819 | ACAGATACTGGACGATCACG | CTGAGTCTGATTCGGCATGT |
| 163 | 18 | 112034\|114003 | 102779\|104747 | 104785\|102816 | SP21-1 | 102816-102870,102905-102945,104253-104394,104729-104785 | CCAACCCTTACGACCAATAGC | GTACACGATTCGGAATGCCC |
| 164 | 18 | 112034\|114003 | 102779\|104747 | 104785\|102816 | SP22-1 | 102816-102870,102905-102945,104253-104394,104729-104785 | CCAACCCTTACGACCAATAGC | GTACACGATTCGGAATGCCC |
| 165 | 157 | 111781\|113573 | 101944\|104317 | 104355\|101490 | SP21-1 | 101490-101577,102813-102870,102917-102944,104252-104355 | CCAACCCTTACGACCAATAGC | GTACACGATTCGGAATGCCC |
| 166 | 157 | 111781\|113573 | 101944\|104317 | 104355\|101490 | SP21-1 | 101490-101577,102813-102870,102905-102944,103787-103890,104252-104355 | CCAACCCTTACGACCAATAGC | GTACACGATTCGGAATGCCC |
| 167 | 158 | 111781\|113573 | 101944\|104317 | 104355\|101490 | SP21-2 | 101490-101755,102813-102879 | AGGTCCATGCCCGAATACAA | GTACACGATTCGGAATGCCC |
| 168 | 159 | 100340\|102141 | 101944\|104317 | 104355\|100267 | SP21-2 | 100267-100490,104303-104355 | AGGTCCATGCCCGAATACAA | GTACACGATTCGGAATGCCC |
| 169 | 160 | 112030\|113612 | 102774\|104356 | 104394\|102811 | SP21-2 | 102811-102870,104303-104394 | AGGTCCATGCCCGAATACAA | GTACACGATTCGGAATGCCC |
| 170 | 161 | 111913\|113636 | 102657\|104380 | 104418\|102694 | SP21-2 | 102694-102870,104303-104418 | AGGTCCATGCCCGAATACAA | GTACACGATTCGGAATGCCC |
| 171 | 158 | 111781\|113573 | 101944\|104317 | 104355\|101490 | SP22 | 101490-101577,102813-102848,104324-104355 | CAAAGCAGAAGTCGTGCAAA | AATCCGGTCTCGGAGTAACC |
| 172 | 158 | 111781\|113573 | 101944\|104317 | 104355\|101490 | SP22 | 101490-101577,102813-102848,104324-104355 | CAAAGCAGAAGTCGTGCAAA | AATCCGGTCTCGGAGTAACC |
| 173 | 158 | 111781\|113573 | 101944\|104317 | 104355\|101490 | SP22 | 101490-101577,102813-102848,104324-104355 | CAAAGCAGAAGTCGTGCAAA | AATCCGGTCTCGGAGTAACC |
| 174 | 158 | 111781\|113573 | 101944\|104317 | 104355\|101490 | SP22 | 101490-101577,102813-102848,104324-104355 | CAAAGCAGAAGTCGTGCAAA | AATCCGGTCTCGGAGTAACC |
| 175 | 162 | 111781\|113612 | 101944\|104356 | 104394\|101008 | SP22 | 101008-101139,102817-102848,104324-104394 | CAAAGCAGAAGTCGTGCAAA | AATCCGGTCTCGGAGTAACC |
| 176 | 163 | 111781\|113612 | 101944\|104356 | 104394\|101010 | SP22 | 101010-101138,102816-102848,104324-104394 | CAAAGCAGAAGTCGTGCAAA | AATCCGGTCTCGGAGTAACC |
| 177 | 164 | 111781\|114003 | 101944\|104747 | 104785\|101008 | SP22 | 101008-101138,102816-102848,104324-104394,104729-104785 | CAAAGCAGAAGTCGTGCAAA | AATCCGGTCTCGGAGTAACC |
| 178 | 165 | 112746\|114004 | 103490\|104748 | 104786\|103527 | SP23 | 103527-103890,104252-104347,104729-104786 | CGATACGAAGACATTTCTCCAC | TGCTATTGGTCGTAAGGGTTG |
| 179 | 18 | 112034\|114003 | 102779\|104747 | 104785\|102816 | SP23 | 102816-102937,104739-104785 | CGATACGAAGACATTTCTCCAC | TGCTATTGGTCGTAAGGGTTG |
| 180 | 18 | 112034\|114003 | 102779\|104747 | 104785\|102816 | SP23 | 102944-102816,104785-104729,104394-104252,103890-103787,102944-102814 | CGATACGAAGACATTTCTCCAC | TGCTATTGGTCGTAAGGGTTG |
| 181 | 18 | 112034\|114003 | 102779\|104747 | 104785\|102816 | SP24 | 102816-102850,102899-102944,103787-103890,104253-104394,104729-104785 | GGGTTCCAGATATACCAACCCT | GCAATCCGGTCTCGGAGTAA |
| 182 | 166 | 111887\|112486 | 102631\|103230 | 103268\|102668 | SP24 | 102668-102849,102899-103268 | GGGTTCCAGATATACCAACCCT | GCAATCCGGTCTCGGAGTAA |
| 183 | 167 | 111984\|112487 | 102728\|103231 | 103269\|102765 | SP24 | 102765-102850,102899-103269 | GGGTTCCAGATATACCAACCCT | GCAATCCGGTCTCGGAGTAA |
| 184 | 168 | 111829\|112265 | 102573\|103009 | 103047\|102610 | SP24 | 102610-102850,102899-103047 | GGGTTCCAGATATACCAACCCT | GCAATCCGGTCTCGGAGTAA |
| 185 | 168 | 111829\|112265 | 102573\|103009 | 103047\|102610 | SP24 | 102610-102850,102899-103047 | GGGTTCCAGATATACCAACCCT | GCAATCCGGTCTCGGAGTAA |
| 186 | 168 | 111829\|112265 | 102573\|103009 | 103047\|102610 | SP24 | 102610-102850,102899-103047 | GGGTTCCAGATATACCAACCCT | GCAATCCGGTCTCGGAGTAA |
| 187 | 169 | 101942\|102528 | 101944\|103031 | 103069\|101713 | SP24 | 101619-101713,102766-102850,102899-103069 | GGGTTCCAGATATACCAACCCT | GCAATCCGGTCTCGGAGTAA |
| 188 | 169 | 101942\|102528 | 101944\|103031 | 103069\|101713 | SP24 | 101619-101713,102766-102850,102899-103069 | GGGTTCCAGATATACCAACCCT | GCAATCCGGTCTCGGAGTAA |
| 189 | 170 | 111985\|112487 | 102729\|103231 | 103269\|102766 | SP24 | 102766-102850,102899-103269 | GGGTTCCAGATATACCAACCCT | GCAATCCGGTCTCGGAGTAA |
| 190 | 170 | 111985\|112487 | 102729\|103231 | 103269\|102766 | SP24 | 102766-102850,102899-103269 | GGGTTCCAGATATACCAACCCT | GCAATCCGGTCTCGGAGTAA |
| 191 | 171 | 111985\|112343 | 102729\|103087 | 103125\|102766 | SP24 | 102766-102850,102899-103125 | GGGTTCCAGATATACCAACCCT | GCAATCCGGTCTCGGAGTAA |
| 192 | 172 | 111781\|113073 | 101944\|103817 | 103855\|101010 | SP24 | 101010-101138,102816-102850,102899-102944,103787-103855 | GGGTTCCAGATATACCAACCCT | GCAATCCGGTCTCGGAGTAA |
| 193 | 173 | 113007\|114003 | 103751\|104747 | 104785\|103788 | SP25-1 | 103788-103892,104687-104785 | CGGGTATACAGCTAAGTGAC | GACCAACCTGCTGTTCGATC |
| 194 | 173 | 113007\|114003 | 103751\|104747 | 104785\|103788 | SP25-1 | 103788-103892,104687-104785 | CGGGTATACAGCTAAGTGAC | GACCAACCTGCTGTTCGATC |
| 195 | 174 | 113401\|113940 | 104145\|104684 | 104722\|104182 | SP25-2 | 104182-104389,104687-104722 | CGGGTATACAGCTAAGTGAC | CCATATGCATGAGCACCGTT |
| 196 | 175 | 113473\|114004 | 104217\|104748 | 104786\|104254 | SP25-2 | 104254-104389,104687-104786 | CGGGTATACAGCTAAGTGAC | CCATATGCATGAGCACCGTT |
| 197 | 176 | 112048\|119093 | 102792\|109837 | 110672\|102829 | SP25-3 | 102829-102881,104687-104786,110614-110672 | CGGGTATACAGCTAAGTGAC | CTGGTAAGTCCGTACACGAT |
| 198 | 176 | 112048\|119093 | 102792\|109837 | 110672\|102829 | SP25-3 | 102829-102881,104687-104786,110614-110672 | CGGGTATACAGCTAAGTGAC | CTGGTAAGTCCGTACACGAT |
| 199 | 18 | 112034\|114003 | 102779\|104747 | 104785\|102816 | SP25-4 | 102816-102881,104256-104394,104729-104785 | GATGGATTGCACTGGACACC | CTGGTAAGTCCGTACACGAT |
| 200 | 18 | 112034\|114003 | 102779\|104747 | 104785\|102816 | SP25-4 | 102816-102881,104256-104517,104731-104785 | GATGGATTGCACTGGACACC | CTGGTAAGTCCGTACACGAT |
| 201 | 18 | 112034\|114003 | 102779\|104747 | 104785\|102816 | SP25-4 | 102816-102881,104256-104517,104731-104785 | GATGGATTGCACTGGACACC | CTGGTAAGTCCGTACACGAT |
| 202 | 177 | 111781\|113996 | 101944\|104740 | 104778\|101513 | SP25-4 | 101513-101577,102813-102881,104256-104394,104729-104778, | GATGGATTGCACTGGACACC | CTGGTAAGTCCGTACACGAT |
| 203 | 178 | 97032\|102141 | 97034\|102143 | 104385\|96959 | SP25-4 | 96959-97027,102814-102881,104385-104256 | GATGGATTGCACTGGACACC | CTGGTAAGTCCGTACACGAT |
| 204 | 18 | 112034\|114003 | 102779\|104747 | 104785\|102816 | SP25-5 | 102816-102881,104429-104517,104731-104785 | CCGCTAATATGGTATCCATGG | CTGGTAAGTCCGTACACGAT |
| 205 | 18 | 112034\|114003 | 102779\|104747 | 104785\|102816 | SP25-5 | 102816-102881,104429-104517,104731-104785 | CCGCTAATATGGTATCCATGG | CTGGTAAGTCCGTACACGAT |
| 206 | 176 | 112048\|119093 | 102792\|109837 | 110672\|102829 | SP25-5 | 110614-110672,102881-102829,104786-104429 | CCGCTAATATGGTATCCATGG | CTGGTAAGTCCGTACACGAT |
| 207 | 179 | 111781\|113736 | 101944\|104480 | 104518\|101528 | SP25-5 | 104429-104518,101528-101577,102813-102881 | CCGCTAATATGGTATCCATGG | CTGGTAAGTCCGTACACGAT |
| 208 | 180 | 111781\|113994 | 101944\|104738 | 104776\|101010 | SP25-5 | 101010-101138,102816-102881,104429-104617,104731-104776 | CCGCTAATATGGTATCCATGG | CTGGTAAGTCCGTACACGAT |
| 209 | 181 | 112730\|113680 | 103474\|104424 | 104462\|103511 | SP25-6 | 103511-103741,104429-104462 | CCGCTAATATGGTATCCATGG | GAGCCACGGATAGAGGAACA |
| 210 | 182 | 112658\|113683 | 103402\|104427 | 104465\|103439 | SP25-6 | 103439-103741,104429-104465 | CCGCTAATATGGTATCCATGG | GAGCCACGGATAGAGGAACA |
| 211 | 183 | 112576\|113701 | 103320\|104445 | 104483\|103357 | SP25-6 | 103357-103741,104429-104483 | CCGCTAATATGGTATCCATGG | GAGCCACGGATAGAGGAACA |
| 212 | 184 | 112523\|113727 | 103267\|104471 | 104509\|103304 | SP25-6 | 103304-103739,104429-104509 | CCGCTAATATGGTATCCATGG | GAGCCACGGATAGAGGAACA |
| 213 | 185 | 112880\|113759 | 103624\|104503 | 104541\|103661 | SP25-6 | 103616-103741,104429-104541 | CCGCTAATATGGTATCCATGG | GAGCCACGGATAGAGGAACA |
| 214 | 186 | 111781\|114003 | 102344\|104747 | 104785\|102381 | SP26 | 104328-104394,104729-104785,102381-102445,102816-102937 | GCTATTGGTCGTAAGGGTTGGAAT | GCAGAAGTCGTGCAAACACG |
| 215 | 18 | 112034\|114003 | 102779\|104747 | 104785\|102816 | SP26 | 102816-102937,104328-104394,104729-104785 | GCTATTGGTCGTAAGGGTTGGAAT | GCAGAAGTCGTGCAAACACG |
| 216 | 18 | 112034\|114003 | 102779\|104747 | 104785\|102816 | SP26 | 102899-102816,104785-104729,104394-104328 | GCTATTGGTCGTAAGGGTTGGAAT | GCAGAAGTCGTGCAAACACG |
| 217 | 187 | 99913\|102141 | 99915\|102143 | 103891\|99840 | SP26-2 | 99840-99908,102382-102445,102816-102937,103833-103891 | GCTATTGGTCGTAAGGGTTGGAAT | CTGTGCAGATTCAATCGAGCC |
| 218 | 188 | 111781\|114014 | 101944\|104758 | 104796\|101058 | SP27 | 101058-101138,102816-102944,104761-104796 | CCCCCTGTAATACCCGTAAA | CTGAGTGTTGCTATTGGTCGTAA |
| 219 | 189 | 127419\|128834 | 114935\|116350 | 116527\|115111 | SP28 | 115111-115215,116460-116527 | CTTTACGCGCACCGATTCAG | ACTCAGGAGAGTTTGTGGCG |
| 220 | 190 | 127390\|129016 | 114906\|116532 | 116709\|115082 | SP28 | 115082-115215,116460-1165281,16553-116709 | CTTTACGCGCACCGATTCAG | ACTCAGGAGAGTTTGTGGCG |
| 221 | 190 | 127390\|129016 | 114906\|116532 | 116709\|115082 | SP28 | 115082-115215,116460-116709 | CTTTACGCGCACCGATTCAG | ACTCAGGAGAGTTTGTGGCG |
| 222 | 191 | 128535\|129243 | 116051\|116759 | 116936\|116227 | SP29 | 116227-116335,116610-116936 | ATGTGGATTGCGCGGAAAAT | CATCGCCGTTTAACGTAGGG |
| 223 | 192 | 128391\|129061 | 115907\|116577 | 116754\|116083 | SP29 | 116083-116337,116610-116754 | ATGTGGATTGCGCGGAAAAT | CATCGCCGTTTAACGTAGGG |
| 224 | 193 | 128616\|129163 | 116132\|116679 | 116856\|116308 | SP29 | 116308-116337,116610-116856 | ATGTGGATTGCGCGGAAAAT | CATCGCCGTTTAACGTAGGG |
| 225 | 194 | 128616\|129208 | 116132\|116724 | 116901\|116308 | SP29 | 116308-116337,116610-116901 | ATGTGGATTGCGCGGAAAAT | CATCGCCGTTTAACGTAGGG |
| 226 | 195 | 128306\|129024 | 115822\|116540 | 116717\|115998 | SP29 | 115998-116337,116610-116717 | ATGTGGATTGCGCGGAAAAT | CATCGCCGTTTAACGTAGGG |
| 227 | 196 | 128469\|129162 | 115985\|116678 | 116855\|116161 | SP29 | 116161-116337,116610-116855 | ATGTGGATTGCGCGGAAAAT | CATCGCCGTTTAACGTAGGG |
| 228 | 197 | 126100\|128981 | 113616\|116497 | 116674\|113792 | SP29 | 113792-113898,116460-116674 | ATGTGGATTGCGCGGAAAAT | CATCGCCGTTTAACGTAGGG |
| 229 | 198 | 128569\|129377 | 116085\|116893 | 117070\|116261 | SP29 | 116261-116337,116610-117070 | ATGTGGATTGCGCGGAAAAT | CATCGCCGTTTAACGTAGGG |
| 230 | 199 | 65491\|102141 | 65493\|102143 | 119494\|65418 | SP30 | 65418-65546,119319-119494 | CGACGCTGTCGCGTTATGAA | CACGGCGAATAACGTGTCTT |
| 231 | 199 | 65491\|102141 | 65493\|102143 | 119494\|65418 | SP30 | 65418-65546,119319-119494 | CGACGCTGTCGCGTTATGAA | CACGGCGAATAACGTGTCTT |
| 232 | 199 | 65491\|102141 | 65493\|102143 | 119494\|65418 | SP30 | 65418-65546,119319-119494 | CGACGCTGTCGCGTTATGAA | CACGGCGAATAACGTGTCTT |
| 233 | 200 | 41431\|102141 | 41431\|102143 | 119492\|494 | SP30 | 494-610,119319-119492 | CGACGCTGTCGCGTTATGAA | CACGGCGAATAACGTGTCTT |
| 234 | 200 | 41431\|102141 | 41431\|102143 | 119492\|494 | SP30 | 494-610,119319-119492 | CGACGCTGTCGCGTTATGAA | CACGGCGAATAACGTGTCTT |
| 235 | 200 | 41431\|102141 | 41431\|102143 | 119492\|494 | SP30 | 494-610,119319-119492 | CGACGCTGTCGCGTTATGAA | CACGGCGAATAACGTGTCTT |
| **Primers for circRNAs qPCR verification** | | | | | | | | |
| Human circRNAs | | hsa_circRTN4_012 | | | | | TTGGTCATGTGAACTGCACG | AGCTGATGGTCACAGAGAGC |
|  |  | hsa_circESYT2_032 | | | | | TGCCAAATGCGTCAAACCTC | GGGGTCTGACTTTCCCTTGA |
|  |  | hsa_circ_chr16_00001 | | | | | TGTGTGGCCTTCTCCATCAC | GGCCTAGAACCCTGGGGAGG |
|  |  | hsa_circCDYL_005 | | | | | AAGCCGGTCGGAGCTTTATT | CTCACAGTTCACGAGGTGCT |
|  |  | hsa_circCYP24A1_012 | | | | | TCCAGGCCACAGACAATGAG | CGTGGCCTCTTTCATCACAG |
|  |  | hsa_circSPECC1_006 | | | | | TGAAAGTAGCCCGAGCAGAG | CGTAGGAGTGGGAGTGTTGG |
| VZV circRNAs | | 41839\|42280 | 41839\|42280 | 42211\|41769 | qSP1 | 41769-41880,42133-42211 | AGTTTGTCAATAGGTCCGTTTGGGG | CATGTCGGCGTATGATCGACAAC |
|  |  | 75877\|77973 | 75879\|77975 | 77901\|75804 | qSP2 | 75804-75854,77827-77901 | ATATCCACCAGTGAAGCTCGATGAC | TACCCGGGGATCCTCTAGAGATTTG |
|  |  | 86080\|86381 | 86082\|86383 | 86309\|86007 | qSP3 | 86007-86084,86224-86309 | GTAATCGCGTAGTTTCCTGTGTCTT | CGATAAATCCACACAACGCCGC |
|  |  | 112035\|114003 | 102779\|104747 | 104785\|102816 | qSP4 | 104406-104517,104731-104785,102816-102944,103787-103890 | GACATTTCTCCACCCCCCTGTAA | GCTATTGGTCGTAAGGGTTGGT |
|  |  | 85376\|85940 | 85378\|85942 | 85868\|85303 | qLP1 | 85770-85868,85303-85372 | ATCCGGTTACTTTCCAGCGCTAAA | TTCTGCAGGGTGGGGATTTAGG |
|  |  | 100293\|100697 | 100295\|100699 | 100625\|100220 | qLP2 | 100551-100625,100218-100311 | GGTGGTGTTATGCCACGTTTTACA | GATGTCATCTGGTGCTAACACACG |
|  |  | 112097\|113995 | 102841\|104739 | 104777\|102878 | qLP3 | 104314-104394,104712-104777,102878-102939 | GAGCGATACGAAGACATTTCTCCAC | TTGCTATTGGTCGTAAGGGTTGGT |
|  |  | 119996\|120396 | 110732\|111132 | 111311\|110910 | qLP4 | 110982-110910,111311-111239 | TAGATAAAGTCGAGGCATATGCGCC | ATCGGACGGGGAAGACTTTATAGT |
| **Experimentally confirmed VZV circRNAs in HZ patients** | | | | | | | | |
| Patients | | **pOka**  **(AB097933.1)** | **Primer**  **name** | **Fragments**  **(AB097933.1)** | | | **Primer F**  **Sequence (5'to3')** | **Primer R**  **Sequence (5'to3')** |
| patient1 | | 77901\|75802 | SP2 | 75802-75854,77827-77901 | | | TGTACAGCATCAGGTCGAAT | TGACGACAGCGCTATATCCA |
| Patient3 | | 77901\|75802 | SP2 | 75802-75854,77827-77901 | | | TGTACAGCATCAGGTCGAAT | TGACGACAGCGCTATATCCA |
| Patient4 | | 77901\|75802 | SP2 | 75802-75854,77827-77901 | | | TGTACAGCATCAGGTCGAAT | TGACGACAGCGCTATATCCA |
| Patient5 | | 77901\|75802 | SP2 | 75802-75854,77827-77901 | | | TGTACAGCATCAGGTCGAAT | TGACGACAGCGCTATATCCA |
| Patient6 | | 77901\|75802 | SP2 | 75802-75854,77827-77901 | | | TGTACAGCATCAGGTCGAAT | TGACGACAGCGCTATATCCA |
| Patient2 | | 86266\|86044 | SP3 | 86044-86084,86215-86266 | | | AACAGAGGACGCCGATAAATCC | GCAATGACCGCAATGCTGAT |
| patient4 | | 86278\|86028 | SP3 | 86028-86084,86222-86278 | | | AACAGAGGACGCCGATAAATCC | GCAATGACCGCAATGCTGAT |
| patient4 | | 86292\|86031 | SP3 | 86031-86084,86222-86292 | | | AACAGAGGACGCCGATAAATCC | GCAATGACCGCAATGCTGAT |
| patient2 | | 86309\|86007 | SP3 | 86007-86084,86224-86309 | | | AACAGAGGACGCCGATAAATCC | GCAATGACCGCAATGCTGAT |
| patient3 | | 86309\|86007 | SP3 | 86007-86084,86224-86309 | | | AACAGAGGACGCCGATAAATCC | GCAATGACCGCAATGCTGAT |
| patient4 | | 86313\|86022 | SP3 | 86022-86084,86215-86313 | | | AACAGAGGACGCCGATAAATCC | GCAATGACCGCAATGCTGAT |
| patient5 | | 86313\|86022 | SP3 | 86022-86084,86215-86313 | | | AACAGAGGACGCCGATAAATCC | GCAATGACCGCAATGCTGAT |
| patient6 | | 86313\|86022 | SP3 | 86022-86084,86215-86313 | | | AACAGAGGACGCCGATAAATCC | GCAATGACCGCAATGCTGAT |
| patient4 | | 86315\|86043 | SP3 | 86043-86084,86215-86315 | | | AACAGAGGACGCCGATAAATCC | GCAATGACCGCAATGCTGAT |
| patient4 | | 86315\|86043 | SP3 | 86215-86315,86043-86084 | | | AACAGAGGACGCCGATAAATCC | GCAATGACCGCAATGCTGAT |
| patient4 | | 86263\|85894 | SP3 | 85894-86084,86222-86263 | | | AACAGAGGACGCCGATAAATCC | GCAATGACCGCAATGCTGAT |
| patient5 | | 86263\|85894 | SP3 | 85894-86084,86222-86263 | | | AACAGAGGACGCCGATAAATCC | GCAATGACCGCAATGCTGAT |
| patient6 | | 86263\|85894 | SP3 | 85894-86084,86222-86263 | | | AACAGAGGACGCCGATAAATCC | GCAATGACCGCAATGCTGAT |
| patient2 | | 104785\|102381 | SP24 | 102381-102445,102816-102870,102905-102944,103787-103890,104252-104394,104729-104785 | | | GGGTTCCAGATATACCAACCCT | GCAATCCGGTCTCGGAGTAA |
| patient2 | | 104785\|102381 | SP24 | 102381-102445,102816-102870,102905-102944,103787-103890,104252-104394,104729-104785 | | | GGGTTCCAGATATACCAACCCT | GCAATCCGGTCTCGGAGTAA |
| patient2 | | 104394\|102378 | SP24 | 102378-102445,102816-102870,102912-102945,104252-104394 | | | GGGTTCCAGATATACCAACCCT | GCAATCCGGTCTCGGAGTAA |
| patient2 | | 102945\|98902 | SP24 | 98902-99077,102399-102445,102816-102870,102905-102945 | | | GGGTTCCAGATATACCAACCCT | GCAATCCGGTCTCGGAGTAA |
| patient2 | | 103850\|93543 | SP24 | 93534-93576,99941-100123,101009-101138,102383-102445,102816-102870,102912-102944,103787-103850 | | | GGGTTCCAGATATACCAACCCT | GCAATCCGGTCTCGGAGTAA |
| patient3 | | 104785\|102381 | SP26 | 104328-104394,104729-104785,102381-102445,102816-102937 | | | GCTATTGGTCGTAAGGGTTGGAAT | GCAGAAGTCGTGCAAACACG |
| patient4 | | 104785\|102381 | SP26 | 104328-104394,104729-104785,102381-102445,102816-102937 | | | GCTATTGGTCGTAAGGGTTGGAAT | GCAGAAGTCGTGCAAACACG |
| patient5 | | 104785\|102381 | SP26 | 104328-104394,104729-104785,102381-102445,102816-102937 | | | GCTATTGGTCGTAAGGGTTGGAAT | GCAGAAGTCGTGCAAACACG |
| patient3 | | 104785\|102816 | SP26 | 102816-102937,104328-104394,104729-104785 | | | GCTATTGGTCGTAAGGGTTGGAAT | GCAGAAGTCGTGCAAACACG |
| Patient4 | | 104785\|102816 | SP26 | 102816-102937,104328-104394,104729-104785 | | | GCTATTGGTCGTAAGGGTTGGAAT | GCAGAAGTCGTGCAAACACG |
| Patient5 | | 104788\|102386 | SP26 | 102386-102445,102816-102937,104328-104394,104729-104788 | | | GCTATTGGTCGTAAGGGTTGGAAT | GCAGAAGTCGTGCAAACACG |
| patient3 | | 103891\|99840 | SP26-2 | 99840-99908,102382-102445,102816-102937,103833-103891 | | | GCTATTGGTCGTAAGGGTTGGAAT | CTGTGCAGATTCAATCGAGCC |
| patient3 | | 104339\|102861 | SP26-2 | 102861-102935,103833-103890,104252-104339 | | | GCTATTGGTCGTAAGGGTTGGAAT | CTGTGCAGATTCAATCGAGCC |
| patient4 | | 104374\|101854 | SP31 | 101854-101943,102377-102445,102816-102863,104280-104374 | | | ATTCGGAATGCCCGCAAT | GAGAGGACACTGGACGCTGG |
| **Convergent primers** | | | | | | | | |
| GAPDH | | | GCACCGTCAAGGCTGAGAAC | | | | TGGTGAAGACGCCAGTGGA | |
| ORF28 | | | AAGCTTTCTGGAGATGCGCT | | | | TGTGGAAAAAGGGTGGCAGT | |
| IE61 | | | ACATCCCTGCGTTGTCTTT | | | | TTGAGGTGGTTTCTGGTCTTA | |
| circVLTs_lytic_ mutagenesis | | | CCAACGAAGACACACCCCTA | | | | ACTTGCATTACCCTATCCCAGT | |
| VLTs_lytic_ | | | CGGATATCAGAGCAGGATGCCCCGGT | | | | TCCCCGCGGTTATTTACGGGTATTACAGGGGGGT | |
| ORF63 | | | CCGGAATTCATGTTTTGCACCTCACCGGC | | | | GAAGATCTCTACACGCCATGGGGGG | |
| **Probes for ampFISH** | | | | | | | | |
| circVLTs_lytic_ (+) DP | | | GTTACAGACGACTCCCACAGTCCTTATTTACGGGTATTACAGGGGGGGGACT | | | | | |
| circVLTs_lytic_ (+) AP | | | GGAGTAACCGGGGCATCCTGCTCTGGACTGTGGGAGTCGTCTGTAACTACTTCATGTTACAGACGACTCCCAC | | | | | |
| circVLTs_lytic_ (-) DP | | | GTTACAGACGACTCCCACAGTCCGAGCAGGATGCCCCGGTTACTCCGGGACT | | | | | |
| circVLTs_lytic_ (-) AP | | | CCCCCTGTAATACCCGTAAATAAAGGACTGTGGGAGTCGTCTGTAACTACTTCATGTTACAGACGACTCCCAC | | | | | |
| VLTs_lytic_ DP linear | | | GTTACAGACGACTCCCACAGTCCCGTGTACGGACTTACCAGGGGGCAGGACT | | | | | |
| VLTs_lytic_ AP linear | | | GACCGGATTGCGGGCATTCCGAATGGACTGTGGGAGTCGTCTGTAACTACTTCATGTTACAGACGACTCCCAC | | | | | |
| **Full-length sequences of circVLTs_lytic_** | | | | | | | | |
| CTTTATTTACGGGTATTACAGGGGGGTGGAGAAATGTCTTCGTATCGCTCTTTATCTGCACCATATGCATGAGCACCGTTTCCGATCTCGGAAAAACCATGCCGTGTTTGCACGACTTCTGCTTTGTTTGTATTCGGGCATGGACCTCCACCAGCGTCCAGTGTCCTCTCTGCCGGTGTCCAGTGCAATCCATCCTGCTGTTCGATCGGCGACCCCGGTGGTGTATGGCTCGATTGAATCTGCACAGCAACCCCGGACCGGGGGGCAGGACTACCGTGATCGTCCAGTATCTGAGTGTTGCTATTGGTCGTAAGGGTTGGTATATCTGGAACCCAAGGTGTAAATACTGCCCCCTGGTAAGTCCGTACACGATTCGGAATGCCCGCAATCCGGTCTCGGAGTAACCGGGGCATCCTGCT | | | | | | | | |
