# Supplementary material for: Identification and characterization of Varicella Zoster Virus circular RNA in lytic infection
Source: Nat Commun. 2024 Jun 10;15:4932. doi: 10.1038/s41467-024-49112-4 (PMC11164961; doi:10.1038/s41467-024-49112-4)

M1-1:114004-119699(131805), 119784(131720)|112109

111-113

TTCTCCACCCCCCTGTAATACCCGTAAATAAAGGGTTCGTCCGATTCATAACGCGACAGCGTCG


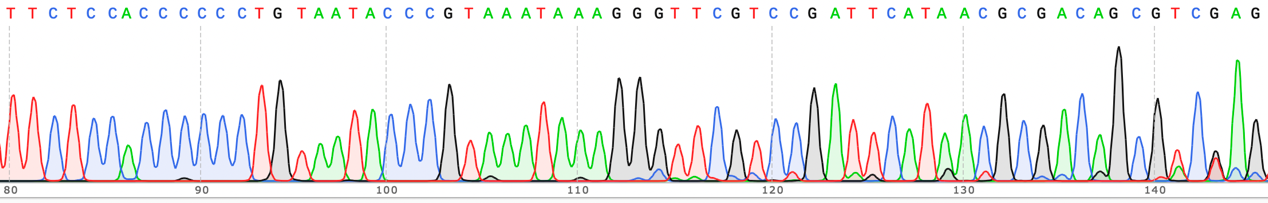


188-196

GAAAAGGTTACTACGGCCCCAAGGACATGTTTTACACCTTGGGTTCCAGATATACCAACCCTTA


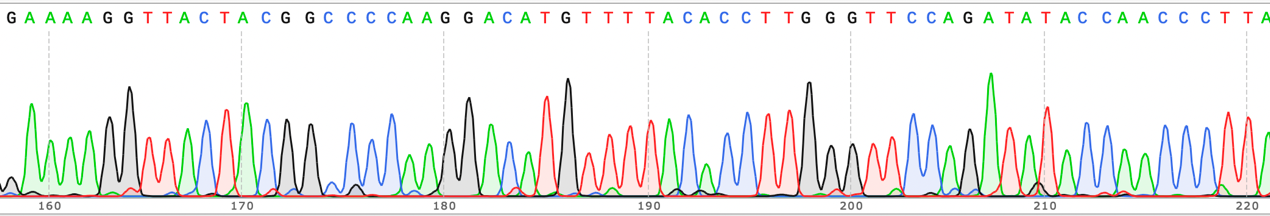


M1-2:113005-112162, 113103|112030

74-76

ATTCCAACCCTTACGACCAATAGCAACACTCAGATACTGGACGATCACGGTAGTCCTGCCCCCC


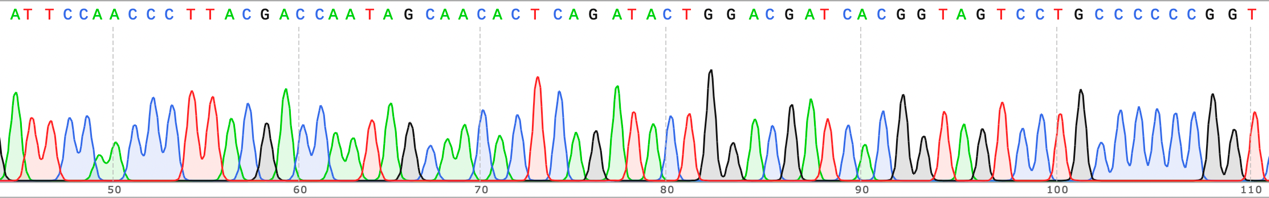


169-172

CATACACCACCGGGGTCGCCGATCGAACAGCAGAGCAGGATGCCCCGGTTACTCCGAGACCG


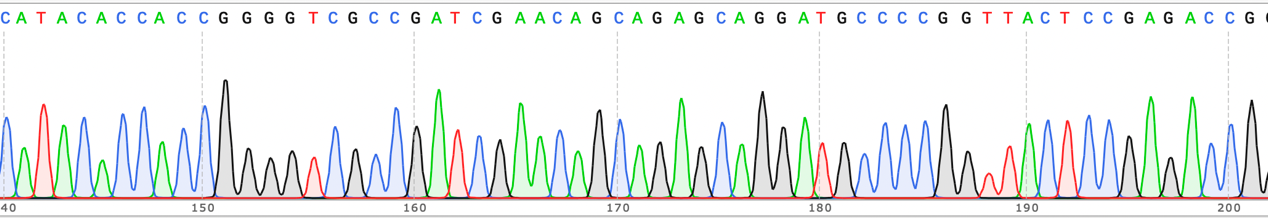


M2-1: 114003|102341, 112027|102402

168-170

CTCGGAGTAACCGGGGCATCCTGCTAGCAAAGGCAACGGTGTTTTCGGGTCAGGCAGCCGG


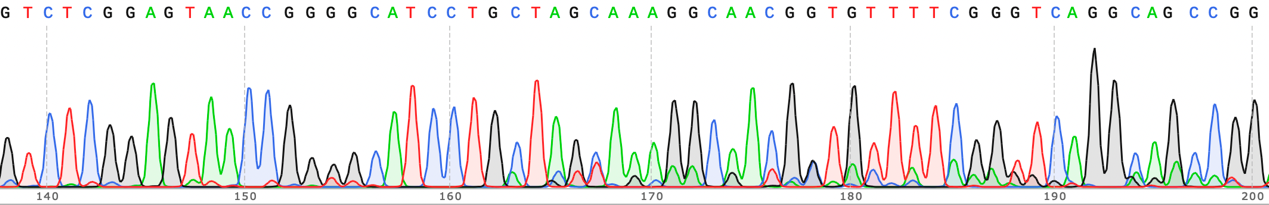


228-229

GACCCGTTTAAAATGCCAACTGGATAGTCTCAGCAACGATCCTACTACAAGGGGTGGAGAA


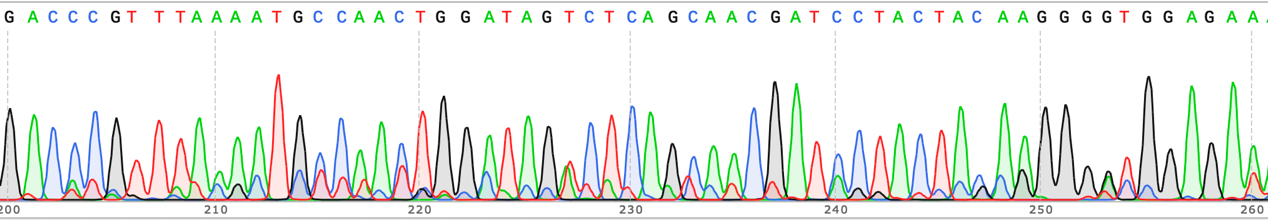


M2-2:114003-114655(136841), 114740(136756)-112121

97-107

GACATTTCTCCACCCCTTCTAGTAGGATCGTTGCTGAGTGCGCCGCTGTCGCACCGCCTGGGTTT


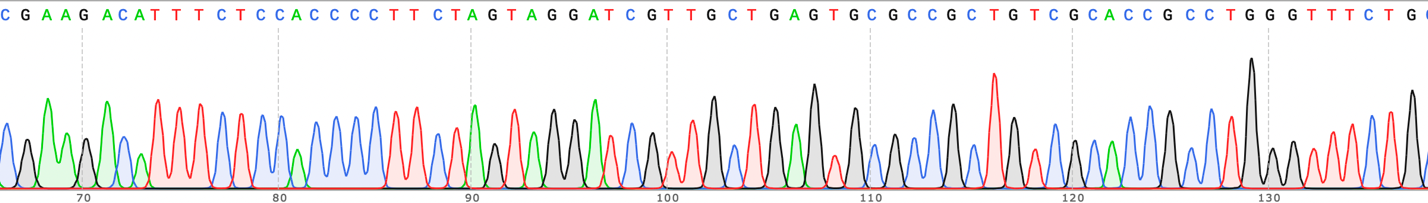


179-183

CCGAGCCCCCGTGGTGTCCGAACACGAACCGTGTTCCAGATATACCAACCCTTACGACCAATA


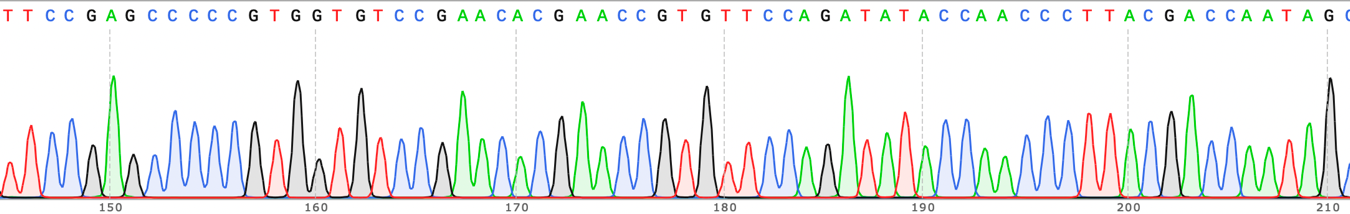


M2-3:114003|119692(131812)

104-113

CGCTGTCGCGTTATGAATCGGACGAACCCTCAGCAACGATCCTACTAGAAGGGGTGGAGAAAT


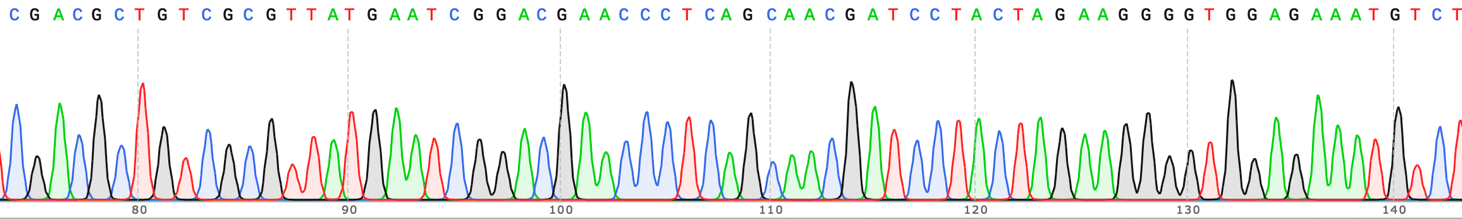


M2-4:113041|112043, 113005-112162

155-157

ATACCAACCCTTACGACCAATAGCAACACTCAGATACTGGACGATCACGGTAGTCCTGCCCCCC


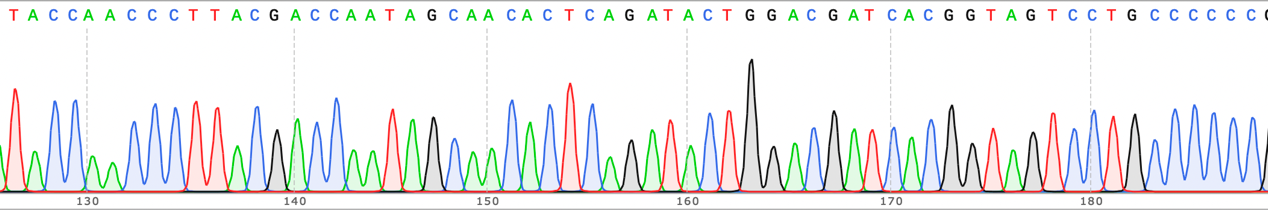


185-191

ATACTGGACGATCACGGTAGTCCTGCCCCCCGGTTACTCCGAGACCGGATTGCGGGCATTCCG


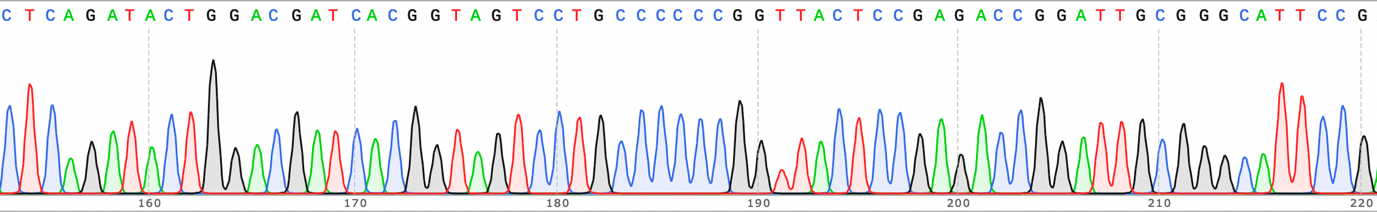


M2-5:112473|111857

377-382

AGATGTCAGGTCTATTGTTAAGCATCGTTTAACGTCTCAACTCGGCCTTGGTTGCATCTCCAAGT


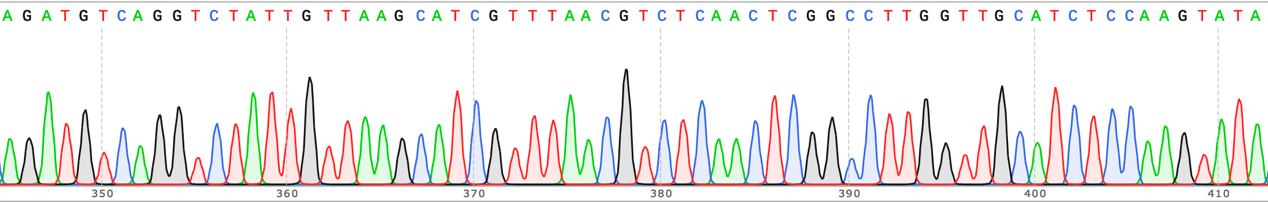


M2-6:113005|112162, 113470|113108, 113947|113612, 114004|119692(131812), 119765(131739)|112042

71-73

GATTCCAACCCTTACGACCAATAGCAACACTCAGATACTGGACGATCACGGTAGTCCTGCC


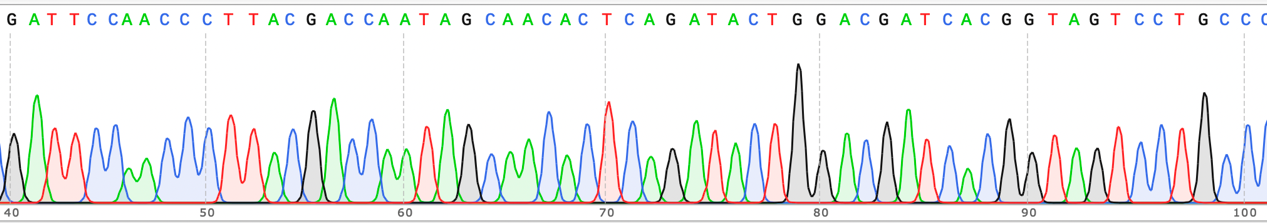


166-174

TACACCACCGGGGTCGCCGATCGAACAGCAGGATGGATTGCACTGGACACCGGCAGAGAGGA


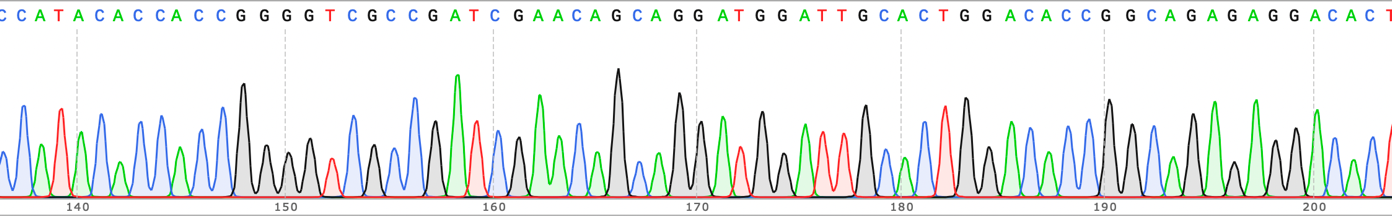


307-308

GATCGGAAACGGTGCTCATGCATATGGTGCAGATAAAGAGCGATACGAAGACATTTCTCCAC


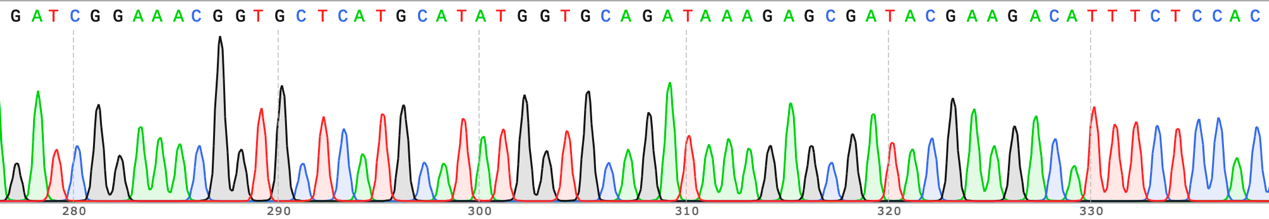


355-364

CATTTCTCCACCCCTTCTAGTAGGATCGTTGCTGAGGGTTCGTCCGATTCATAACGCGACA


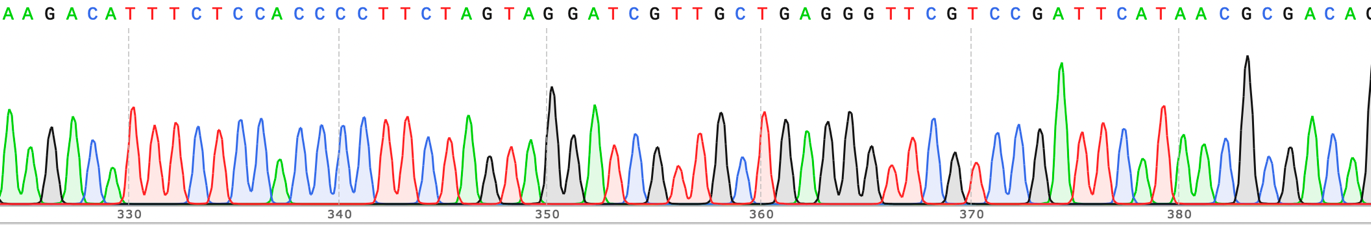


424-428

GAGTCGGTTTTAAGGGAAAAGATTACTACGGCCCCGGTTACTCCGAGACCGGATTGCGGGCATT


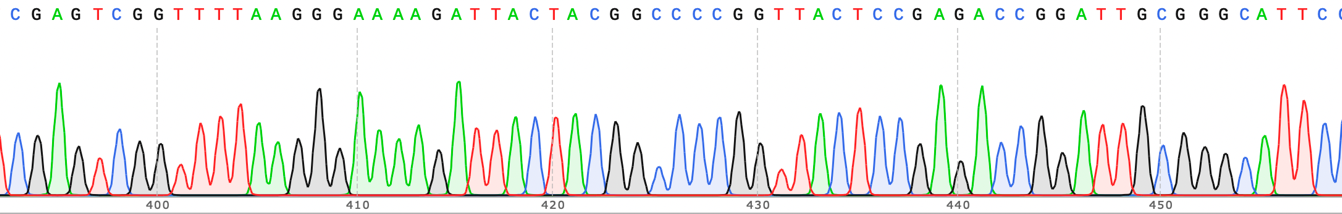


M2-7:112162-113005, 113103|112023, 112162-113005, 113159|111872,

69-71

TTCCAACCCTTACGACCAATAGCAACACTCAGATACTGGACGATCACGGTAGTCCTGCCCC


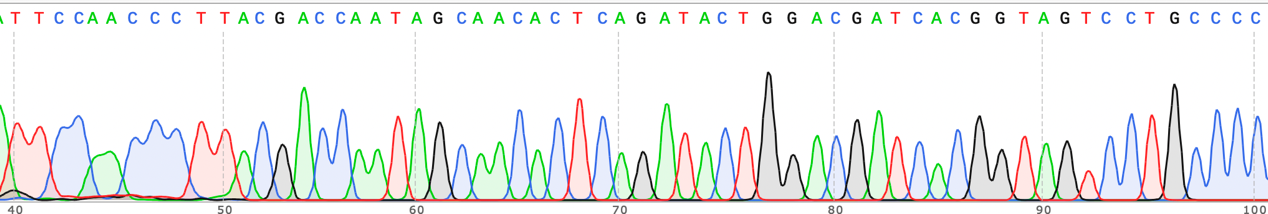


164-167

ACACCACCGGGGTCGCCGATCGAACAGCAGAGCAGGATGCCCCGGTTACTCCGAGACCGG
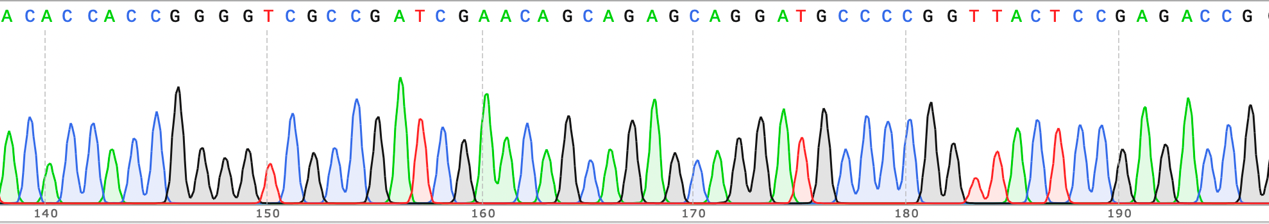


294-296

TACCAACCCTTACGACCAATAGCAACACTCAGATACTGGACGATCACGGTAGTCCTGCCCCCC


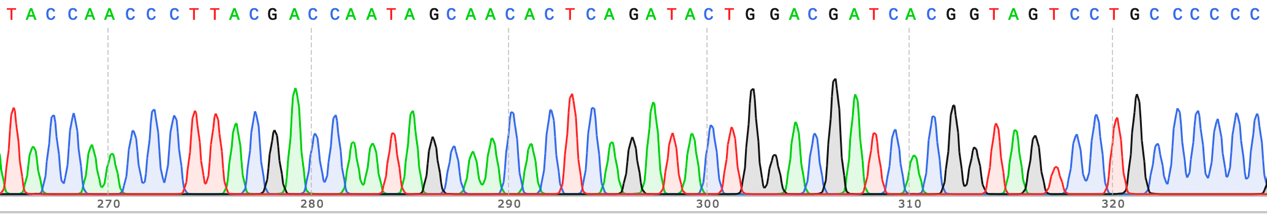


443-448

TTCCGTAAAAATGATGCGGTAGAGCATGTTTTGGTTGCATCTCCAAGTATACCTTTAGTTTGCTCC


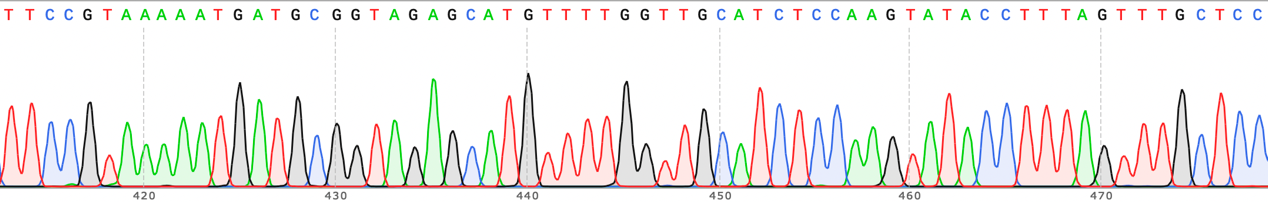


M2-8:112163|112069

152

CTGGTAAGTCCGTACACGATTCGGAATGCCCCTGGGTGTTGCTATTGGTCGTAAGGGTTGGT


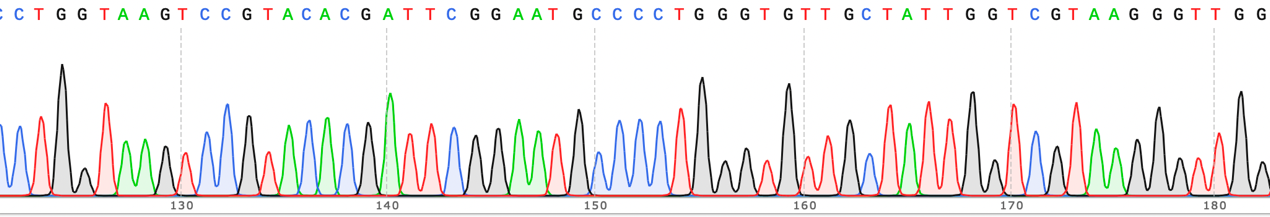


M2-9:112163|112069, 112153|112067

157

CCAACCCTTACGACCAATAGCAACACCCAGGGGCATTCCGAATCGTGTACAGACTTGCCAG


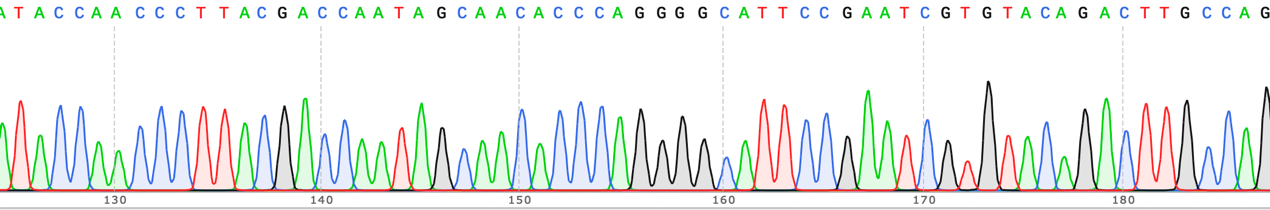


241-242

GTTCCAGATATACCAACCCTTACGACCAATAGCGCGGGCATTCCGAATCGTGTACAGACTTGCC


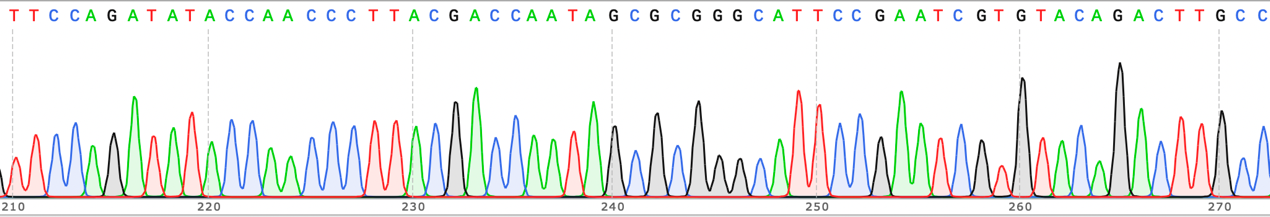


M2-10:112153|112067

371-372

CTGGTAAGTCTGTACACGATTCGGAATGCCCGCGCTATTGGTCGTAAGGGTTGGTATATCTGG


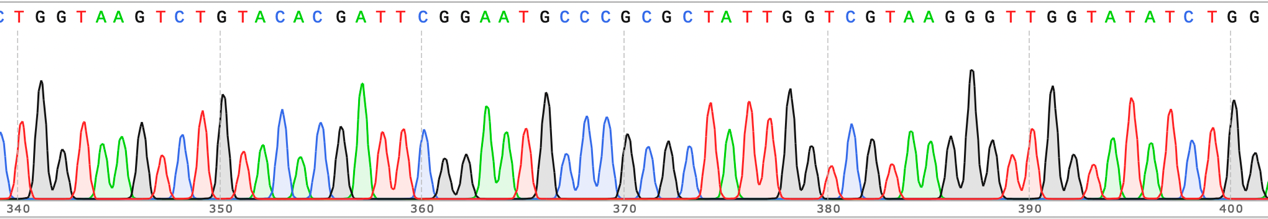

Supplement: Supplementary file 7 — Supplementary data 4 [file 41467_2024_49112_MOESM7_ESM.docx]
